# Deep generative virtual staining for breast cancer: from H&E histology to HER2 immunohistochemistry

**DOI:** 10.1186/s12880-026-02834-x

**Published:** 2026-10-31

**Authors:** Asmaa Yaser, Samar Elbedwehy, Azza Abdel-Aziz, Abeer Twakol Khalil

**Affiliations:** 1 Department of Biomedical Engineering, Faculty of Engineering, Mansoura University, Mansoura, Egypt; 2 Department of Information Technology, Faculty of Computers and Artificial Intelligence, Damietta University, Damietta, 34517 Egypt; 3 Department of Computer Science, Faculty of Artificial Intelligence and Informatics, Horus University, Damietta, Egypt; 4 Pathology Department, Faculty of Medicine, Mansoura University, Mansoura, Egypt; 5 Department of Electronics and Communications Engineering, Faculty of Engineering, Mansoura University, Mansoura, Egypt

**Keywords:** Image-to-image translation, Virtual IHC staining, Breast histopathology, Conditional GAN, Diffusion-based image translation, Multi-objective optimization, Integrated gradients, Explainable AI (XAI)

## Abstract

Immunohistochemistry (IHC) remains the clinical gold standard for molecular profiling in breast cancer; however, traditional workflows are hindered by high costs, reagent dependency, and tissue exhaustion. Virtual staining has emerged as a computational alternative; however, prevailing virtual staining models often fail to preserve high-frequency structural details critical for perceptual and histological fidelity. We present a novel chemically-informed, two-stage conditional Generative Adversarial Network (cGAN) framework designed for high-fidelity H&E-to-IHC translation. Our approach uniquely decouples chemical stain extraction from high-resolution textural refinement through a specialized StainMapper (G₁) and a score-conditioned RefineGenerator (G₂). By integrating multi-scale hierarchical score conditioning with a hybrid loss function including adversarial, perceptual, Sobel edge, and stain-consistency terms, the framework enforces both global distributional realism and local morphological precision. Evaluated on the BCI and HER2match benchmarks, our model achieves state-of-the-art performance, reaching a Fréchet Inception Distance (FID) of **59.67**, PSNR of **22.50 dB**, SSIM of **0.4773**, LPIPS of **0.5279** and KID of **0.0264 ± 0.0086** on BCI; PSNR of **20.66 dB**, LPIPS of **0.4486**, FID of **20.42**, and KID of **0.0124 ± 0.0092** on HER2match, outperforming all baselines on distributional and perceptual metrics. These results demonstrate that incorporating histopathological priors and structural attention mechanisms improves the histological and perceptual fidelity of virtual IHC synthesis, providing a methodological foundation for potential secondary applications such as stain standardization, dataset augmentation, and pathology education in settings where HER2 score is already known.

## Introduction

Cancer remains a leading cause of global mortality [1], with breast cancer (BC) being the most prevalent malignancy among women worldwide [2]. In 2022 alone, approximately 2.3 million new cases and 670,000 deaths were recorded, with a disproportionate impact on low- and middle-income regions [3]. In the United States, BC incidence continues to rise annually, driven largely by hormone receptor-positive cases [2]. As a highly heterogeneous disease, BC requires precise molecular profiling to guide clinical intervention [4].

Histopathology remains the gold standard for cancer diagnosis, revealing critical information about cellular morphology and tissue architecture [5]. While Hematoxylin and Eosin (H&E) staining serves as the fundamental technique for visualizing tissue structure, it lacks the molecular specificity required to distinguish proteomic expressions [1, 5]. Consequently, Immunohistochemistry (IHC) is employed to detect specific biomarkers including, ER, PR, Ki67, and HER2, through targeted antigen-antibody interactions [1]. Approximately 20–25% of BC cases are HER2-positive, a subtype characterized by aggressive clinical progression that necessitates targeted therapies such as Herceptin. Accurate HER2 scoring on a four-point scale (0, 1+, 2+, 3+) is therefore critical, as misclassification can lead to sub-optimal treatment decisions and adverse patient outcomes [6, 7].

Traditional IHC workflows are labor-intensive, reagent-costly, and prone to significant inter-observer variability [2, 8]. Practical challenges such as tissue exhaustion from repeated sectioning, the absence of standardized staining protocols, and lengthy processing pipelines frequently delay clinical decision-making [1, 6]. To address these systemic bottlenecks, **virtual staining** has emerged as a transformative computational strategy [9]. Formulated as an image-to-image translation task, this approach leverages Deep Generative Models (DGMs) to synthesize IHC maps directly from H&E whole-slide images [3]. Architectures spanning Generative Adversarial Networks (GANs), including conditional GANs (cGANs) and CycleGAN, as well as Contrastive Unpaired Translation (CUT) and Diffusion Models, have demonstrated substantial promise in replicating histological texture and biomarker patterns [3]. By computationally reproducing stains, laboratories can reduce operational costs, preserve finite tissue samples, and accelerate diagnostic turnaround times [3, 6].

Despite these advances, prevailing virtual staining approaches treat the H&E-to-IHC transition as a generic colorization problem, failing to preserve high-frequency structural biomarkers such as cellular membrane completeness, the primary morphological criterion for HER2 scoring. Recent cross-domain medical imaging studies have shown that introducing intermediate representations between source and target modalities can reduce distribution shift and preserve semantic structure [10], suggesting that a direct jump from H&E to IHC may be suboptimal. In parallel, modality-aligned learning paradigms demonstrate that explicitly modeling correspondence between heterogeneous inputs at the instance or patch level improves downstream predictive reliability [11], suggesting that virtual staining frameworks may benefit from explicit cross-stain alignment beyond conventional pixel-wise objectives. Such cross-stain alignment is particularly important because real-world clinical datasets are often affected by spatial misalignment between consecutive tissue sections, making precise pixel-to-pixel correspondence inherently unreliable and increasing the risk of systematic diagnostic hallucinations during unconstrained generation. Moreover, existing models typically lack the capacity to explicitly condition synthesis on the target clinical score, thereby precluding controllable and interpretable IHC generation. In addition, natural class imbalance across HER2 scores further complicates robust model learning. Unlike conventional image-to-image translation tasks, H&E and HER2 immunohistochemistry (IHC) originate from distinct staining mechanisms and therefore encode complementary **chemical information** about tissue composition. Hematoxylin preferentially binds to nucleic acids, highlighting nuclear morphology, whereas HER2 IHC employs antibody-mediated chromogenic deposition of Diaminobenzidine (DAB) to visualize membrane-bound HER2 protein expression. Consequently, the transformation from H&E to HER2 IHC is not solely a change in color appearance but also a transition between stain-specific representations that reflect different biological characteristics of the tissue [12]. In this work, we refer to this stain-specific information encoded by hematoxylin and DAB as **chemical information**, and use it as an intermediate supervisory representation to guide image synthesis rather than relying exclusively on direct RGB translation. This representation is grounded in the Beer–Lambert law of light absorption, whereby the optical density of a stained specimen is approximately proportional to chromogen concentration and optical path length. Because the optical densities of multiple co-located chromogens combine additively in optical-density space, Hematoxylin and DAB contributions can be approximately linearly separated using the Ruifrok–Johnston stain-deconvolution matrix, yielding physically meaningful stain-component representations that guide the proposed two-stage framework [13]. To address these limitations, we propose a **structurally-aware and score-conditioned generative framework** that explicitly decouples chemical stain extraction from high-resolution IHC refinement, enabling controllable, biologically plausible, and perceptually faithful IHC synthesis.

### The main contributions of this study are summarized as follows

- **Chemically-Informed Two-Stage Translation Architecture**: A two-stage GAN first predicts intermediate hematoxylin and DAB stain-component representations before synthesizing the final HER2 IHC image. These stain-specific representations provide chemically informed guidance during image synthesis and reduce the appearance gap between H&E morphology and HER2 expression.
- **Multi-Scale Hierarchical Score Conditioning**: HER2 score labels are injected at encoder, bottleneck, and decoder levels, preventing label forgetting and enabling controllable IHC generation across the full 0–3 + clinical spectrum.
- **Structural Integrity via Attention-Alignment Synergy**: CBAM attention, Feature Alignment Block (FAB), and stochastic noise injection jointly enforce structural coherence and mitigate the impact of tissue sectioning artifacts.
- **Feature-Anchored Multi-Objective Optimization**: A loss function combining Sobel edge, DAB consistency, perceptual, and adversarial objectives enforces structural and perceptual fidelity at both pixel and feature levels.
- **Bias-Aware Training for Clinical Score Distribution**: Weighted Random Sampling corrects the skewed clinical HER2 score distribution, ensuring balanced training proficiency from Score 0 through Score 3+.

## Related work

The development of virtual immunohistochemical staining from H&E images has advanced rapidly since 2022, evolving from early pixel-driven GANs toward semantics-aware, registration-robust, contrastive and diffusion-based frameworks. This section provides a structured review of the major contributions and highlights the field-wide limitations that motivate the present work.

### GAN-based translation

Liu et al. [14] established the foundational benchmark by releasing the BCI dataset and proposing Pyramid Pix2pix, a conditional GAN utilizing a ResNet-9 generator with Gaussian multi-scale L1 and cGAN losses for direct H&E-to-IHC translation (PSNR: 21.16 dB, SSIM: 0.477). Despite its benchmark-setting performance, the method suffers from persistent pixel-level misalignments arising from adjacent-slice registration and exhibits severe mode collapse in high-expression (Score 3+) cases. Rehmat et al. [7] subsequently extended this architecture with a batch-wise variance-penalty term to mitigate mode collapse (PSNR: 22.16 dB, SSIM: 0.47, FID: 346.37), though at the cost of increased training complexity and a risk of over-smoothing cytoplasmic textures critical for HER2 scoring.

Huang et al. [15] pursued unpaired translation via an enhanced CycleGAN with explicit texture-preserving constraints and cycle-consistency losses (PSNR: 17.86 dB, SSIM: 0.410). While offering flexibility in data curation, the unsupervised formulation amplified hallucinations in low-contrast tissue regions, underscoring the inherent tension between domain flexibility and biomarker fidelity. Saad et al. [2] integrated conditional generation with downstream HER2 staging in IHC-GAN, using a dual-scale Pix2PixHD conditioned on clinical stage labels (PSNR: 22.87 dB, SSIM: 0.374, FID: 93.0, staging accuracy: 96%). However, forced 256 × 256 downsampling discarded fine structural details essential for membrane scoring. Riccio et al. [6] introduced nuclear-focused spatial weight maps in HEMERA to modulate GAN losses (PSNR: 21.67 dB, SSIM: 0.355), improving downstream segmentation Dice scores yet sacrificing cytoplasmic texture fidelity critical for HER2 interpretation.

### Contrastive and semantics-aware approaches

Li et al. [16] introduced Adaptive Supervised PatchNCE (ASP) loss with progressive scheduling, dynamically weighting patch-level contrastive terms by alignment confidence (BCI SSIM: 0.5032, FID: 65.1). Although effective on misaligned pairs, the method required careful hyper-parameter tuning and imposed significant computational overhead from patch-wise losses, limiting whole-slide scalability. Zhang et al. [17] advanced high-resolution translation with PPT, integrating FocalNCE contrastive loss and 4 × 4 patch-alignment L1 in a pyramid generator focused on nuclear and membrane structures (FID: 52.52, SSIM: 0.4968), though residual ghosting from adjacent-slice misalignment persisted. Jia et al. [8] tackled inconsistent pairs through wavelet-based multi-frequency feature extraction and InfoNCE contrastive objectives (PSNR: 21.38, SSIM: 0.504, FID: 94.1), but the resulting model complexity limited clinical scalability.

### Biologically-informed and decoupled generation

Wei et al. [18] introduced DeReStainer, which decouples the Hematoxylin channel prior to HER2-conditioned DAB generation with SimAM attention (PSNR: 20.65 dB, SSIM: 0.564, semantic top-1 accuracy: 0.563). While pioneering the stain-decoupling concept, the framework was restricted to DAB-based IHC and remained sensitive to registration variance in the channel separation step. Chen et al. [19] proposed PSPStain, employing Protein-Aware Learning Strategies (PALS) with focal optical density maps and Prototype-Consistent Learning (PCLS) to guide biologically plausible staining despite misalignment, achieving the highest biomarker correlation reported to date (Pearson-R: 0.8303 on MIST-HER2). However, this approach required curated prototype banks from pathologist annotations and was untested on non-HER2 biomarkers.

### Diffusion models and same-slide validation

Klöckner et al. [9] introduced the HER2match same-slide re-staining dataset and conducted a rigorous benchmark comparing GANs against diffusion models under perfectly aligned conditions. Among the methods evaluated on HER2match, the top-performing GAN-based entry was BCIStainer, originally reported by Zhu et al. [20] as the leading submission in the BCI challenge; GANs consistently outperformed diffusion models on SSIM, PSNR (PSNR: 22.93 dB, SSIM: 0.559) and FID/KID, but both paradigms failed to reach full clinical utility thresholds. Teixeira et al. [4] proposed CSSP2P, a conditional GAN with cross-scale structural similarity constraints, evaluated on HER2match. The method achieved a pathologist indistinguishability rate of 63.6%, with synthetic images rated at FID 33, KID 0.43 and LPIPS 0.46. These results highlight the persistent gap between pixel-level metrics and clinical judgment, even under perfect registration conditions.

Großkopf et al. [5] proposed HistDiST, a latent diffusion model employing dual-conditioning on H&E morphology via Phikon embeddings and HER2 labels within a stochastic sampling framework (FID: 31.3, SSIM:0.228, Mean Relative Accuracy [MRA]: +28% over baselines on MIST-ER), though slow inference limited practical clinical deployment. Liu et al. [21] reframed synthesis as image restoration with Star-Diff, achieving state-of-the-art PSNR of 21.30 dB and Semantic Fidelity Score (SFS) of 0.74 on BCI, but requiring complex path-balance tuning across staining protocols.

### Field-wide limitations and research gap

As synthesized by Zhu et al. [20] in their formal review of the BCI challenge, persistent limitations across these approaches include: (i) registration artifacts from adjacent-section pairing, (ii) poor correlation between standard pixel metrics (PSNR/SSIM) and clinical HER2 grading accuracy, and (iii) biomarker-specific mode collapse particularly in high-expression Score 3 + cases. Table 1 provides a comprehensive summary of representative prior works along these dimensions. These unresolved challenges directly motivate the present study’s focus on chemically-informed, score-conditioned, and structurally-aware synthesis, with explicit stain-deconvolution priors and hierarchical conditioning to improve both distributional realism and high-frequency edge preservation.

**Table 1:** Summary of representative prior work in virtual IHC staining from H&E images

Author	Year	Datasets	Method	Results	Limitations
Liu et al. [14]	2022	BCI	Pyramid Pix2pix cGAN + Gaussian pyramid multi-scale L1	PSNR: 21.16 dB | SSIM: 0.477 (Pathologist acc.: 37.5–40%)	Mode collapse (Score 3+); adjacent-slice misalignment
Li et al. [16]	2023	BCI, MIST	ASP-PatchNCE + adaptive weighting + ResNet-6 GAN on inconsistent pairs	BCI: SSIM: 0.5032 | FID: 65.1 | KID: 0.0099 MIST: superior on HER2/ER/Ki67/PR	required careful hyper-parameter tuning; high computational overhead
Huang et al. [15]	2023	BCI	CycleGAN + texture-preserving constraints	PSNR: 17.86 dB | SSIM: 0.410	Hallucinated structures in low-contrast regions; misalignment sensitivity
Zhu et al. [20]	2023	BCI	BCI challenge review + top-5 method benchmarking	Best: PSNR: 22.93 dB | SSIM: 0.559 (BCIStainer)	Registration artifacts and metric-clinical mismatch.
Zhang et al. [17]	2024	BCI, HIT-PAX5	PPT GAN + FocalNCE bidirectional contrastive + patch-alignment L1 in pyramid GAN	BCI: PSNR: 19.09 dB | SSIM: 0.4968 | FID: 52.52 HIT-PAX5: SSIM: 0.3548 | PSNR: 17.41 | FID: 80.71 (+ 25.86% pathologist acc.)	Residual ghosting from adjacent-slice misalignment
Wei et al. [18]	2024	BCI	Decoupled H-channel (HED) + DAB generation + SimAM attention	PSNR: 20.65 dB | SSIM: 0.564 | semantic top-1: 0.563	DAB-only; sensitive to registration variance affects structure.
Chen et al. [19]	2024	BCI, MIST	PSPStain + PALS (Focal OD maps) + PCLS prototype consistency	BCI: PSNR: 18.62 | SSIM: 0.450 | Pearson-R: 0.755 MIST-HER2: PSNR: 14.19 | SSIM: 0.188 | Pearson-R: 0.8303	Required curated prototype banks from pathologist annotations; untested on non-HER2 biomarkers.
Jia et al. [8]	2025	BCI, MIST	VMFE wavelet fusion + contrastive encoders + cross-attention	BCI: PSNR: 21.38 | SSIM: 0.504 | FID: 94.1 MIST: best FID on ER	Higher FID on some biomarkers; added model complexity limits scalability.
Saad et al. [2]	2025	BCI	IHC-GAN (dual-scale Pix2pixHD + HER2 conditioning)	PSNR: 22.87 dB | SSIM: 0.374 | FID: 93.0| staging acc.: 96%	Forced 256 × 256 downsampling loses details
Riccio et al. [6]	2025	ACROBAT, BCI	HEMERA(Pyramid Pix2pix + nuclear weight map + segmentation supervision)	PSNR: 21.67 dB | SSIM: 0.355 Seg: F1: 0.631 | IoU: 0.487	Nuclear focus sacrifices texture (low SSIM); lower recall from imbalance.
Klöckner et al. [9]	2025	HER2match, BCI	Benchmark of 3 GANs vs. 3 DMs on same-slide aligned pairs	GANs (BCIstainer) outperform DMs on SSIM/PSNR/FID	Slow DM inference; insufficient biomarker pattern accuracy.
Rehmat et al. [7]	2025	BCI	Batch variance-penalty on Pyramid Pix2pix	PSNR: 22.16 | SSIM: 0.47 | FID: 346.37	Required paired data; added training complexity; risk of over-smoothing
Großkopf et al. [5]	2025	MIST, BCI	LDM with Phikon embeddings + H&E latent conditioning + stochastic schedule	MIST-ER: SSIM: 0.228 | FID: 31.3 | MRA: +28% BCI: superior vs. all GANs	Inversion limits diversity; slow inference limited practical clinical deployment
Liu et al. [21]	2025	BCI	Star-Diff (Dual-path restoration diffusion + structure-aware saliency)	PSNR: 21.30 dB | SSIM: 0.53 | SFS: 0.74	Complex path-balance tuning; limited pathologist validation
Teixeira et al. [4]	2025	HER2match	CSSP2P cGAN + CSS structural similarity on HER2match	FID: 33 | KID: 0.43 | LPIPS: 0.46 | Pathologist indistinguishability rate: 63.6%	Traditional metrics fail due to re-staining artifacts; smaller dataset.
Ours	2026	BCI, HER2match	Two-stage cGAN + stain-deconvolution priors+ multi-scale HER2 score conditioning + Multi-Scale Hinge Discriminator	BCI: PSNR: 22.50 | SSIM: 0.4773 | LPIPS: 0.5279 | FID: 59.67 | KID: 0.0264 ± 0.0086; **HER2match**: **PSNR: 20.66 dB | SSIM: 0.4467 | LPIPS: 0.4486 | FID: 20.42 | KID: 0.0124 ± 0.0092**	Generalizability to non-HER2 biomarkers not yet validated; used with paired data only.

## Proposed methodology

**Fig. 1:**
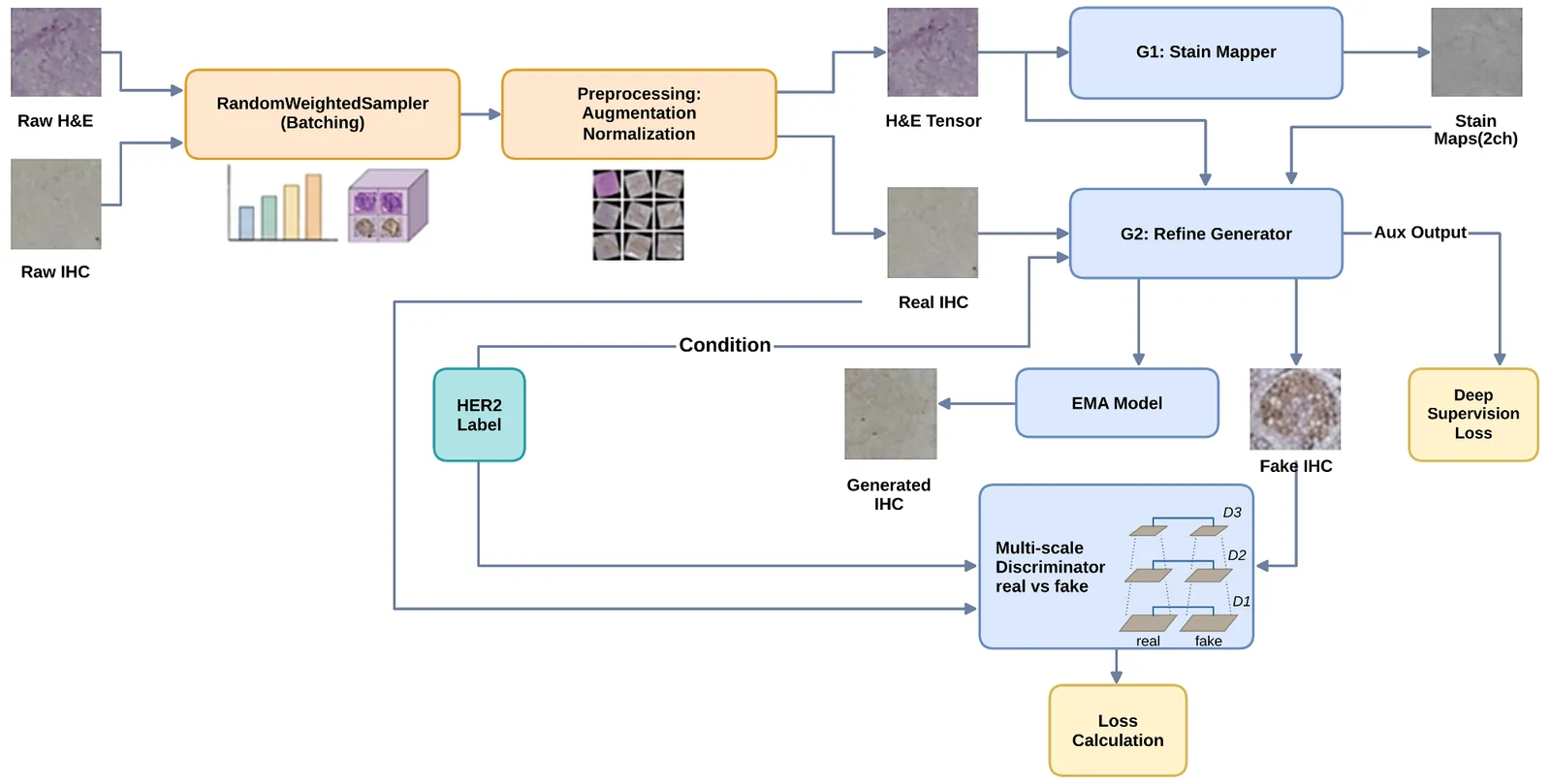
Overview of the proposed two-stage cGAN framework. The StainMapper (G₁) extracts Hematoxylin and DAB stain priors from the H&E input. The RefineGenerator (G₂) synthesizes the final IHC image conditioned on the stain prior, the original H&E, and the target HER2 score embedding. The Multi-Scale Discriminator (D) evaluates authenticity and score consistency at three spatial scales

The proposed framework instantiates a two-stage conditional Generative Adversarial Network (cGAN) architecture, illustrated schematically in Fig. 1, and designed for high-fidelity H&E-to-IHC translation with explicit focus on HER2-expression-pattern synthesis conditioned on known biomarker status. The design is predicated on two architectural principles that are not jointly exploited in conventional single-stage virtual staining frameworks: (i) the physical decoupling of stain chemistry from appearance synthesis, and (ii) the hierarchical injection of clinical-score information throughout the generation pathway.

In Stage 1, the **StainMapper (G₁)**, a U-Net variant augmented with an Efficient Attention mechanism and a weighted residual projection path, predicts intermediate Hematoxylin (H) and Diaminobenzidine (DAB) stain-component maps using supervision derived from optical-density-based stain decomposition of the reference IHC images, following the Beer–Lambert-grounded stain deconvolution described in Section “Introduction”. These stain-component maps preserve stain-specific information associated with nuclear morphology and HER2 chromogenic expression, respectively, and serve as a chemically informed structural prior for the subsequent image refinement stage rather than relying solely on implicit feature representations learned during end-to-end translation. Because Hematoxylin and DAB correspond to distinct staining components with different histological roles, predicting these intermediate stain maps provides a stain-specific representation before appearance refinement, thereby reducing the modality gap between H&E morphology and HER2 chromogenic expression.

In Stage 2, the **RefineGenerator (G₂)**, a deep ResNet-based architecture incorporating nine CBAM-integrated residual blocks, stochastic noise injection, and a Feature Alignment Block (FAB), synthesizes the final virtual IHC image from the original H&E image, the predicted stain-prior maps, and a target HER2 score embedding injected at three hierarchical levels. The predicted stain-component maps constrain the refinement generator through two complementary mechanisms. First, they are concatenated with the original H&E image to form the input of G₂, ensuring that stain-specific chemical information is explicitly available throughout feature extraction rather than requiring the network to infer it solely from RGB appearance. Second, the refinement generator is optimized using a hybrid objective that includes reconstruction, perceptual, adversarial, and stain-related consistency losses on the synthesized IHC image. During optimization, the network learns to exploit the stain-prior channels because they provide informative guidance for minimizing these objectives, thereby reinforcing stain-consistent synthesis without imposing explicit supervision on the predicted stain maps themselves. Together, these architectural and optimization constraints guide the generator toward stain-consistent image synthesis while preserving tissue morphology.

Adversarial supervision is provided by a **Conditional Multi-Scale Discriminator (D)** operating concurrently across three spatial resolutions (512 × 512, 256 × 256, and 128 × 128 pixels) under a Hinge loss objective.

The complete generator training objective comprises 13 complementary loss terms spanning adversarial, feature-matching, pixel-level reconstruction, structural, perceptual, stain-space, and regularization objectives. The discriminator is trained under a separate Hinge adversarial objective, with generator and discriminator weights updated in alternating steps. G₁ and G₂ share a single optimizer and are trained end-to-end.

The framework is evaluated against four representative baselines drawn from the principal architectural paradigms in virtual IHC staining. Pix2pix [14] employs a U-Net generator coupled with a PatchGAN discriminator, trained under a composite L1 and adversarial objective. PyramidPix2pix [14] extends this with a multi-scale Gaussian pyramid generator. ASP [16] leverages a ResNet-6 GAN trained with an Adaptive Supervised PatchNCE contrastive loss for handling inconsistent ground-truth image pairs. PSPStain [19] incorporates focal optical density maps and a Prototype-Consistent Learning for semantics-preserving generation. All baselines were retrained from scratch on identical data splits at 512 × 512 resolution under equivalent hardware conditions to ensure statistically unconfounded comparison.

### Stage 1: Chemically-informed stain mapper (*G1*)

**Fig. 2:**
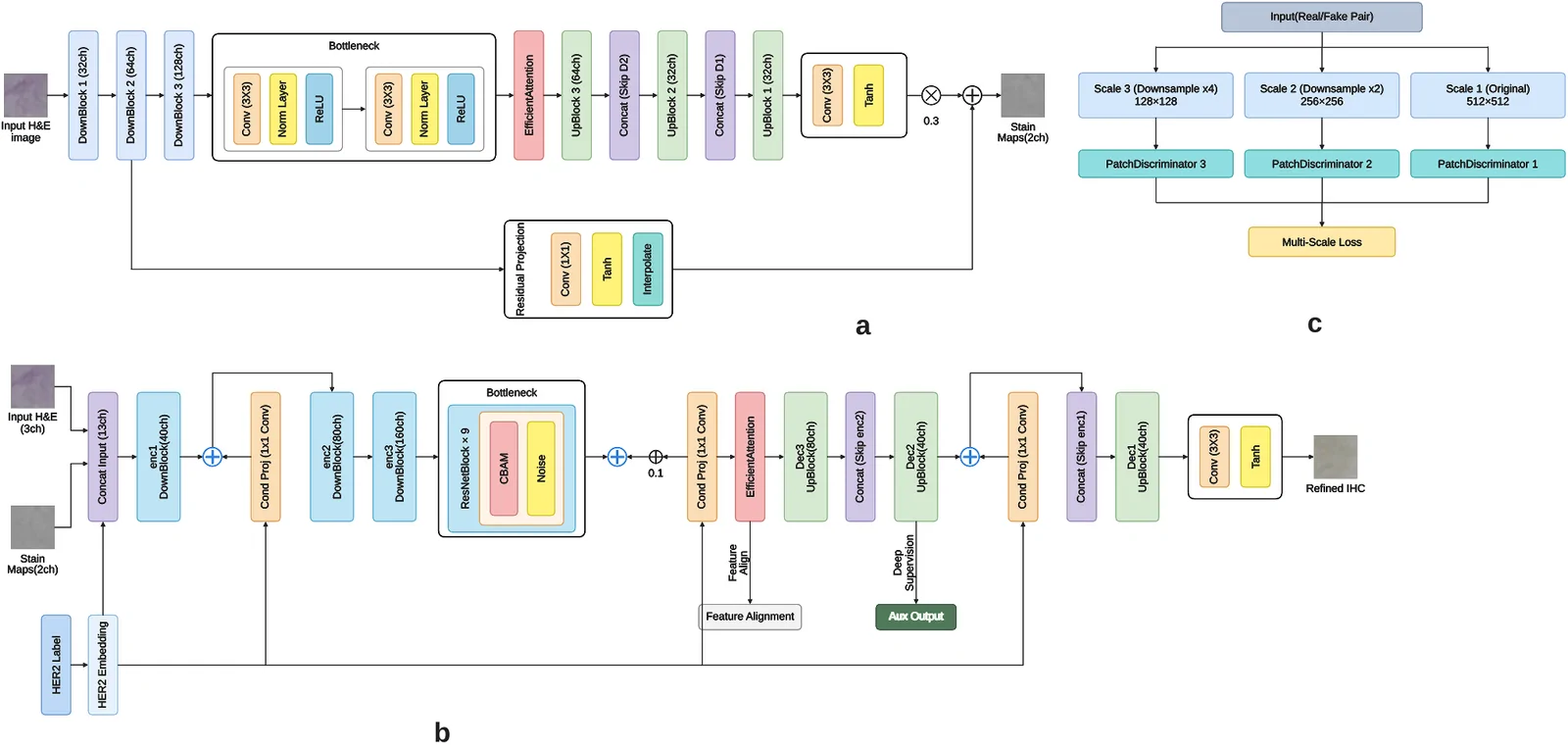
Architecture of the three core components. (**a**) StainMapper G₁: a U-Net with Efficient Attention and residual projection. (**b**) RefineGenerator G₂: a ResNet-9 architecture with CBAM attention, stochastic noise injection, Feature Alignment Block (FAB), and multi-scale HER2 score conditioning. (**c**) Multi-Scale Discriminator D: three parallel PatchGAN branches operating at 512 × 256 × 128. resolution

To address the under constrained nature of direct RGB-to-RGB chromatic translation, the framework initiates with a dedicated U-Net-based generator [13], $\:G_1$, depicted in Fig. 2(a). Rather than relying on unconstrained color mapping, this module learns to predict Hematoxylin and DAB distributions, with supervision provided by classical optical density deconvolution of the ground-truth IHC images via Ruifrok–Johnston matrix [22]. Given an input H&E image $\:{I}_{H\&E}\in\:{R}^{3\times\:H\times\:W}$, $\:G_1$ processes the raw pixels through three progressively deeper DownBlocks (32, 64, and 128 channels) with a symmetric upsampling path to directly produce a two-channel stain representation:

1 $$\:\widehat{S}={G}_{1}\left({I}_{H\&E}\right)\:,\:\widehat{S}\in\:{R}^{2\times\:H\times\:W}$$

where $\:\widehat{S}\:$encodes the spatially-resolved distributions of the isolated Hematoxylin (H) channel, associated with nuclear and stromal staining, and the Diaminobenzidine (DAB) channel, encoding HER2 membrane expression via the brown chromogenic precipitate. The intermediate representation $\:\widehat{S}$ constitutes a chemically-informed prior that guides Stage 2 synthesis by providing explicit stain-chemistry channels alongside the raw H&E input.

High-frequency structural fidelity is preserved through two mechanisms: an **Efficient Attention block** at the network bottleneck, which performs lightweight channel recalibration via adaptive pooling and 1D convolution [23]; and a **weighted** output combination that fuses the UNet **pathway** (scaled by 0.3) with a learned input-to-output projection, providing a spatial shortcut that mitigates detail loss across the compression–expansion hierarchy.

### Stage 2: Refine generator (*G2*)

The core synthesis component of the framework is the RefineGenerator ($\:G_2$), illustrated in Fig. 2(b), which receives a concatenated 5-channel input tensor formed by the channel-wise fusion of the H&E image and the stain prior predicted by $\:G_1$:

2 $$\:{x}_{in}=\left[{I}_{H\&E}\:;\widehat{S}\:\right]\in\:{R}^{5\times\:H\times\:W}$$

where $\>\>[{\rm{\cdot}}\>;\>{\rm{\cdot}}]$ denotes channel-wise concatenation along the feature dimension, $\:{I}_{H\&E}\in\:{R}^{3\times\:H\times\:W}\:$ is the original H&E image, and $\:\widehat{S}\in\:{R}^{2\times\:H\times\:W}$ is the two-channel stain prior from$\:\:G_1$. This multi-modal input design grounds the subsequent synthesis simultaneously in raw tissue morphology and the predicted stain-component representation, providing G₂ with complementary morphological and stain-specific computational information throughout the refinement process rather than relying solely on RGB appearance. Controllable, score-conditioned synthesis is enabled through hierarchical **multi-scale injection of the target HER2 score label** $\:y\in\:\:\left\{\mathrm{0,1}+,2+,3+\right\}$, first mapped to a continuous embedding vector via a learnable embedding table and subsequently projected into the generator latent space at three distinct additive injection levels, in addition to the input-level concatenation through score-specific 1 × 1 convolutional projections [20, 21]:

3 $$\:{c}_{k}={Proj}_{k}\left(Embedding\right(y\left)\right),\:k\in\:\left\{enc2,\:botle,\:dec2\right\}$$

where * k* indexes the three injection levels, encoder (* enc2*), bottleneck (* botle*), and decoder (* dec2*), and $\:{c}_{k}\in\:{\mathbb{R}}^{\mathbb{d}}$ is the resulting conditioning vector projected into the feature space of the corresponding level via a learned $\:1\times\:1$ convolutional projection. This hierarchical conditioning strategy maintains the HER2 score information throughout the synthesis pathway by reinforcing the conditioning signal at multiple network depths, allowing score-specific information to influence feature representations from coarse structural layouts to fine chromogenic details.

The encoder extracts hierarchical multi-scale representations through successive DownBlocks. At the $\:enc2\:\:$level, the conditioning vector $\:{c}_{enc2\:\:}$ is injected via a $\:1\times\:1$ Conditional Projection layer to modulate the global stylistic attributes of the feature representations in accordance with the target HER2 phenotype. The bottleneck architecture integrates three synergistic modules that collectively enforce structural fidelity and synthesis quality under noisy conditions. First, within each residual block of the bottleneck stack, a **Convolutional Block Attention Module (CBAM)** [24] infers channel and spatial attention maps in sequence to selectively amplify synthesis-relevant feature activations:

4 $$\:{F}^{{\prime\:}}={M}_{c}\left(F\right)\otimes\:F$$

5 $$\:{F}^{{\prime\:}{\prime\:}}={M}_{s}\left({F}^{{\prime\:}}\right)\otimes\:{F}^{{\prime\:}}$$

where $\:F\in\:{\mathbb{R}}^{C\times\:H\times\:W}$ is the bottleneck feature tensor; $\:{M}_{c}\left(F\right)\in\:{\mathbb{R}}^{C\times\:1\times\:1}$ is the channel attention map computed via global average-pooling and max-pooling followed by a shared two-layer MLP, encoding inter-channel dependencies; $\:{M}_{s}\left({F}^{{\prime\:}}\right)\in\:{\mathbb{R}}^{1\times\:H\times\:W}$ is the spatial attention map obtained by channel-pooling followed by a $\:7\times\:7$ convolution, encoding intra-channel spatial saliency; and $\:\otimes\:$ denotes element-wise multiplication. The sequential application of channel and spatial attention enhances informative feature responses while suppressing less relevant activations, improving the representation of spatial and contextual information during image synthesis.

Second, within each residual block, lightweight stochastic **noise injection** introduces localized textural variation through a learnable scaled Gaussian noise map [25]. A spatial Gaussian noise map $\:\epsilon\:\sim\:N\left(0,I\right)$ is sampled per block, and modulated by a learnable per-channel scaling parameter α before being added to the convolutional output within each residual unit:

$$\:{F}_{\mathrm{noise}}=F+\alpha\:\odot\:\epsilon\:\:\:\:\:\:\:\:\:\:\:\:\:\:\:\:\:\:\:\left(6\right)$$

where * F*is the bottleneck feature tensor. This lightweight perturbation reduces deterministic over-smoothing while encouraging realistic microscopic texture generation, without interfering with the hierarchical HER2 score conditioning applied throughout the network.

After the complete residual stack — where CBAM attention and stochastic noise injection are applied within each of the nine constituent blocks as described above — the dampened bottleneck conditioning vector $\:{c}_{botle\:}$is added to the resulting feature tensor $\:B\in\:{\mathbb{R}}^{C\times\:H\times\:W}$, followed by an **Efficient Attention module** that performs lightweight channel-wise recalibration via adaptive average pooling, a one-dimensional convolution (kernel size = 3), and sigmoid gating, yielding the gated bottleneck representation $\:{B}_{gate}$.

Third, a **Feature Alignment Block (FAB)** [26] projects $\:{B}_{gate}\:$into a compact embedding space for patch-level contrastive supervision rather than performing explicit spatial alignment. The block applies a 3 × 3 convolution followed by instance normalization and a ReLU activation, then a 1 × 1 convolution with a second instance normalization, before applying channel-wise L2 normalization. The resulting embedding is defined as:

7 $$\:\begin{array}{cccc}&\:z=\mathrm{L}2\;\mathrm{N}\mathrm{o}\mathrm{r}\mathrm{m}\left(f\left({B}_{gate}\right)\right)\end{array}$$

where $\:{B}_{gate}$ denotes the bottleneck feature map after the residual stack, additive HER2-score conditioning, and Efficient Attention gating; $\:f(\cdot\:)$ represents the projection head composed of successive convolutional and normalization layers; and * z* is the resulting 128-dimensional L2-normalized embedding. These embeddings are used exclusively for the patch-level contrastive objective, encouraging synthesized features to remain close to their corresponding real IHC features in the learned representation space while preserving structural consistency [26].

During the upsampling phase, the decoder additionally receives the score conditioning vector $\:{c}_{dec2\:\:}$ at the $\:dec2\:$ level via a Conditional Projection layer, guiding the reconstruction of fine-grained chromogenic texture and structural details. An auxiliary decoding branch at $\:dec2\:$ generates an intermediate output $\:{\hat{I}}_{aux}\:$ for deep supervision [27], providing an additional gradient pathway that facilitates optimization during early training and improves gradient propagation across the decoder.

The final IHC output is produced by a terminal convolutional layer followed by Tanh activation, mapping the synthesized IHC image to the normalized intensity range [-1,1].

### Stage 3: Conditional multi-scale discriminator ($\boldsymbol{D}$)

To simultaneously enforce high-fidelity photorealistic synthesis and HER2-status-aware adversarial realism, a Conditional Multi-Scale Discriminator (*D*) is employed, illustrated in Fig. 2(c). The architecture follows a multi-layer PatchGAN design [28], in which each discriminator branch evaluates the realism of spatially localized image patches rather than the image globally, providing high-density adversarial supervision that is particularly sensitive to fine-grained chromogenic and structural artifacts. The discriminator operates concurrently across three spatial scales: Scale 1 at the native $\:512\times\:512$ resolution; Scale 2 on downsampled $\:256\times\:256\:(\times\:2)$ inputs; and Scale 3 on $\:128\times\:128\:(\times\:4)$ inputs. Each scale corresponds to an independent PatchDiscriminator branch that outputs a 2D patch-confidence map. The multi-scale discriminator objective aggregates the losses from the three discriminator branches by averaging their individual contributions, encouraging the generator to preserve structural integrity and chromogenic consistency across both global tissue organization and local cellular detail.

Score consistency is enforced by spatially broadcasting the embedded HER2 label and concatenating it channel-wise with the H&E input and the corresponding real or generated IHC image at every discriminator scale. This enables the discriminator to perform label-conditioned realism assessment, ensuring generated images are indistinguishable from real IHC images of the corresponding HER2 expression level. Within each PatchDiscriminator branch, spectral normalization on convolutional weights stabilizes training by constraining the Lipschitz constant of the discriminator, while instance normalization stabilizes intermediate feature statistics during adversarial training. The discriminator is optimized under a multi-scale Hinge adversarial objective (Section  “Multi-objective optimization and loss function”). During generator optimization, intermediate feature representations extracted from the discriminator are additionally used to compute a multi-scale feature-matching loss, providing complementary supervision that encourages the generated images to match the hierarchical feature statistics of real IHC images.

### Multi-objective optimization and loss function

Joint optimization of $\:G_1$ and $\:G_2\:$ requires a training objective that enforces fidelity simultaneously across multiple biological and perceptual dimensions. A naive pixel-wise regression loss is known to be insufficient for histopathological image synthesis, producing over-smoothed outputs in which the fine-grained chromogenic heterogeneity and high-frequency structural detail required for reliable IHC scoring are systematically suppressed [14, 28]. Accordingly, the total generator loss $\:{\mathcal{L}}_{\mathrm{G}}$ is formulated as a weighted linear combination of 13 complementary terms, each encoding a distinct inductive bias grounded in the biological and perceptual properties of IHC-stained tissue:

8 $$\:{\mathcal{L}}_{G}\:=\:{\sum\:}_{i\:\in\:S}{\lambda\:}_{i}{\mathcal{L}}_{i}$$

$$\eqalign{& \>S\> = \>\{ adv,\>FM,\>L1,\>edge,\>SSIM,\>LPIPS \cr & ,\>DAB1,\>H1,\>DAB2,\>ds,\>color,\>contr,\>var\} \cr}$$

where * S* is the set of 13 constituent loss terms and $\:\left\{\lambda\:i\right\}$ are scalar weighting coefficients that govern the relative contribution of each term according to the two-phase annealing schedule detailed in the Loss Weight Schedule subsection. Both $\:G_1$ and $\:G_2$ are optimized jointly through $\:{\mathcal{L}}_{\mathrm{G}}\:$: terms$\:\:{\mathcal{L}}_{DAB1}$ and $\:{\mathcal{L}}_{H1}$ supervise $\:G_1$ directly via its stain-map output, while all remaining terms operate on the full-resolution IHC output of $\:G_2$. Loss computation occurs at the end of each forward pass through the complete two-stage network, enabling end-to-end gradient propagation through both generators.

#### Adversarial and feature-matching losses

The adversarial component follows the multi-scale Hinge formulation, wherein the generator minimizes the negative expected discriminator response [29]:

9 $$\:{\mathcal{L}}_{adv}\:=\:-\frac{1}{K}\sum\:_{k=1}^{K}\mathbb{E}\left[{D}_{k}\right(x,\widehat{y}\left)\right]$$

where *K* = 3 is the number of discriminator scales, $\:x\:=\:{I}_{H\&E}\:$is the H&E input, and $\>\hat y\> = \>{G_2}\left( {{x_{in}}} \right)$ is the generated IHC image. This objective incentivizes the generator to produce IHC images that maximally deceive the discriminator across all three spatial resolutions. To supplement the sparse adversarial gradient signal, particularly during early training when the discriminator rapidly achieves saturation, a feature-matching loss is additionally employed [30], penalizing statistical discrepancies between intermediate discriminator activations computed on real and generated images:

10 $$\begin{aligned}{\mathcal{L}_{FM}} &= \mathbb{E}\Bigg[ \sum\nolimits_{k = 1} {^3} \sum\nolimits_i {\left( {1/{N_i}} \right)} \cr&\quad\left\| {{D_k}^{(i)}\left( {{I_{target}}} \right) - {D_k}^{(i)}\left( {G\left( {{x_{in}}} \right)} \right)} \right\|{ _1} \Bigg]\end{aligned}$$

where $\>D_k^{\left( i \right)}({\rm{\cdot}})$ denotes the activation at the * i-th* intermediate layer of the * k-th* discriminator scale; $\:{I}_{target}\:$ is the ground-truth IHC image; and * Ni* is the number of elements in the corresponding feature map. By operating in the discriminator’s learned feature space, $\:{\mathcal{L}}_{FM}$ provides a dense, semantically-rich supervision signal that stabilizes adversarial training and prevents gradient vanishing during the warm-up phase of optimization.

#### Pixel-level reconstruction losses

Low-level pixel fidelity is enforced through a class-weighted * L1* reconstruction term that simultaneously penalizes absolute synthesis errors and corrects for the skewed clinical HER2 score distribution in the training data [28]:

11 $${\mathcal{L}_{L1}} = \mathbb{E}\left[ { w\left( c \right) \cdot{{\left\| {y - \hat y} \right\|}_1} } \right]$$

where $\>{\rm{y}}\> \in \>\>{{\rm{R}}^{{\rm{3 \times H \times W}}}}$ is the ground-truth IHC image, $\>{\rm{\hat y}}\> \in \>\>{{\rm{R}}^{{\rm{3 \times H \times W}}}}$ is the generated IHC image, and $\:w\left(c\right)$ is a per-sample scalar weight assigned according to the inverse frequency of the sample’s HER2 class $\:c\:\in\:\:\{0,\:1+,\:2+,\:3+\}$ within the training distribution. Class-weighted * L1* loss ensures that minority score categories—particularly the diagnostically critical Scores * 2+* and $\:3+\:$, receive proportionally amplified gradient signal, counteracting the majority-class bias that would otherwise result in collapsed synthesis for underrepresented scores. Structural coherence at the level of luminance, contrast, and spatial layout is additionally enforced through the SSIM-derived loss [31]:

12 $$\>{\mathcal{L}_{ssim}}\> = \>1\> - \>SSIM(y,\>\hat y)$$

where $\:SSIM(y,\hat y)\:\in\:\:[0,\:1]$ is the Structural Similarity Index Measure computed locally across sliding windows with stabilizing constants $\:c_1$ and $\:c_2$ (see Section “Performance evaluation metrics” for the full formulation). Minimizing $\:{\mathcal{L}}_{ssim}$ maximizes the perceptual structural correspondence between generated and real IHC images, with particular sensitivity to membrane boundary preservation and local chromogenic contrast.

#### Sobel edge loss

The perimeter-complete membrane staining pattern required for accurate HER2 Score * 2+* and * 3+* scoring constitutes a high-frequency morphological signature that is inadequately captured by pixel-wise or structural losses alone. To provide explicit gradient signal for the preservation of these diagnostically critical boundaries, a Sobel edge loss is introduced. The Sobel gradient magnitude is computed independently for each color channel *C* of an image * I* as [32]:

13 $$\:{\nabla\:}_{S}(I{)}_{C}=\sqrt{({K}_{x}*{I}_{C}{)}^{2}+({K}_{y}*{I}_{C}{)}^{2}+\:\varepsilon\:}$$

where $\:{K}_{x}$ and$\:{\:K}_{y}$ are the fixed $\:3\times\:3$ horizontal and vertical Sobel finite-difference kernels;  * denotes two-dimensional discrete convolution; and $\:\epsilon\:\:>\:0$ is a numerical stability constant that prevents the gradient from becoming undefined at regions of zero intensity variation. The resulting edge loss penalizes the * L1* discrepancy between the Sobel gradient magnitude maps of the real and generated IHC images [32]:

14 $$\:{\mathcal{L}}_{edge}\:=\:\mathbb{E}\mathbb{\:}[\parallel\:{\nabla\:}_{s}\left(y\right)-\:{\nabla\:}_{s}\left(\widehat{y}\right){\parallel\:}_{1}]$$

where $\:{\nabla\:}_{s}\left(y\right)$ and $\:{\nabla\:}_{s}\left(\widehat{y}\right)$ are the channel-averaged Sobel gradient magnitude images of the ground-truth and synthesized IHC images, respectively. This objective penalizes both blurred membrane boundaries and spurious high-frequency chromogenic artifacts, and is shown by ablation (Table 4) to be the single most critical loss term for Score * 2+* and $\:3+\:$synthesis fidelity: its removal raises FID by + 47.5%, as neither $\:{\mathcal{L}}_{L1}$ nor $\:{\mathcal{L}}_{LPIPS}$ alone provides sufficient gradient resolution for preserving high-frequency membrane boundaries.

#### Perceptual loss

High-level textural realism and chromogenic plausibility, properties that are systematically invisible to pixel-level metrics yet highly salient to experienced pathologists, are enforced through the Learned Perceptual Image Patch Similarity (LPIPS) objective [33], which measures the perceptual distance between images in the deep feature space of a pre-trained VGG-16 network:

15 $$\:{\mathcal{L}}_{\mathrm{LPIPS}}=\mathrm{LPIPS}\left(y,\widehat{y}\right)$$

where LPIPS(·) denotes the Learned Perceptual Image Patch Similarity distance computed using a frozen pre-trained VGG-based feature extractor. The LPIPS loss encodes perceptual loss in a way that correlates more strongly with human visual judgments of image quality than PSNR or SSIM [33], making it particularly appropriate for virtual IHC synthesis where perceptual realism is a critical indicator of synthesis quality.

#### Stain deconvolution losses

In addition to pixel-level and perceptual objectives, stain-space supervision is imposed on the predicted hematoxylin and DAB channels obtained through optical-density-based stain decomposition of the reference IHC images. These losses encourage consistency between the synthesized and reference stain distributions, providing chemically informed constraints that complement conventional appearance-based objectives.

Three complementary terms enforce biological fidelity in the HED (Hematoxylin–Eosin–DAB) stain space, obtained by applying the Ruifrok–Johnston optical density color deconvolution matrix $\>{M^{ - 1}}$ [13] to the log-transformed RGB representation of each image. These terms are applied at two stages of the architecture to provide multi-scale stain-space supervision. The first-stage generator $\:G_1$ is supervised directly on its low-resolution stain-channel predictions:

16 $$\>{\mathcal{L}_{DAB1}}\> = {\left\| {{{\hat D}_{low}} - \>{D_{low}}} \right\|_1}$$

17 $$\>{\mathcal{L}_{H1}}\>\>\> = {\left\| {{{\hat H}_{low}}\> - \>{H_{low}}} \right\|_1}$$

where$\:{\widehat{D}}_{low}$ and $\:{D}_{low}$ denote the predicted and ground-truth low-resolution DAB channel images, respectively, extracted via stain deconvolution from the $\:G_1$ output and from the downsampled IHC ground truth; and $\:\:{\widehat{H}}_{low}\:$and $\:{H}_{low}$ denote the corresponding Hematoxylin channel images. These terms supervise the stain mapper directly and encourage physically meaningful stain decomposition. A complementary full-resolution DAB consistency term is additionally applied to the G₂ output, enforcing stain-space fidelity at the spatial resolution of the final synthesized image [7]:

18 $$\>{\mathcal{L}_{DAB2}}\> = {\left\| {{{\hat D}_{full}} - \>{D_{full}}} \right\|_1}$$

where $\:{\widehat{D}}_{full}\:$and $\:{D}_{full}\:$are the DAB channel images extracted via stain deconvolution from *\hat y* and$\:\:y$ at full resolution, respectively. Collectively, $\:{\mathcal{L}}_{DAB1}$, $\:{\mathcal{L}}_{H1}$, and $\:{\mathcal{L}}_{DAB2}$ constrain the DAB chromogenic signal, which directly encodes HER2 membrane overexpression in the diaminobenzidine precipitate—at both spatial scales, linking the generative training objective to a physically interpretable and clinically meaningful stain-space representation.

#### Deep supervision, color, contrastive, and variance losses

An auxiliary output branch at the * dec2* decoder level provides a supplementary gradient pathway that mitigates vanishing gradients in early training epochs [27]:

19 $$\>{\mathcal{L}_{ds}}\> = {\left\| {{{\hat y}_{aux}} - \> \downarrow \>\left( y \right)} \right\|_1}$$

where $\:{\widehat{y}}_{aux}\in\:{\mathbb{R}}^{3\times\:\frac{H}{4}\times\:\frac{W}{4}}$ is the intermediate IHC output of the auxiliary branch, and $\> \downarrow \>({\rm{\cdot}})$ denotes bilinear downsampling of the full-resolution ground truth * y* to the corresponding spatial resolution. This deep supervision term provides an additional stable structural training signal at an intermediate scale, complementing the full-resolution $\:{\mathcal{L}}_{G}\:$objective. The perceptual color loss $\:{\mathcal{L}}_{color\:\:}$enforces global chromatic distribution fidelity by comparing the mean and standard deviation of the generated and reference images across three spatial scales (full, half, and quarter resolution) in RGB space [12]. By matching these global color statistics, the loss penalizes systematic chromatic drift while complementing the localized pixel-level supervision. The contrastive loss $\:{\mathcal{L}}_{contr}\:\:$operates on the L2-normalized embeddings produced by the Feature Alignment Block, encouraging corresponding patches from generated and real IHC images to remain close in the learned representation space while separating non-matching patches through an InfoNCE objective [34]. A variance regularization term suppresses mode collapse and the synthesis of pathologically uniform, structure-deficient outputs [7]:

20 $$\:{\mathcal{L}}_{var}=\mathrm{ReLU}\left({\uptau\:}-{\mathrm{Var}}_{\mathrm{batch}}\left(\widehat{y}\right)\cdot\:t\right)$$

where $\:\tau\:\:\:$is the target variance and $\:t\:$is a scaling factor. This batch-level regularization discourages mode collapse by maintaining sufficient diversity across generated samples throughout training [7].

#### Discriminator loss

The discriminator is optimized under a multi-scale Hinge adversarial objective, computed at each training iteration and used to update the discriminator parameters in alternation with the generator. The loss is defined as [29]:

21 $$\eqalign{& \>{\mathcal{L}_D} = {1 \over K}\sum {\>_{k = 1}^K} \left[ {\mathbb{E}[{\rm{max}}({\rm{0,1}} - {D_k}(x,y))]} \right. \cr & \,\,\,\,\,\,\,\,\,\,\left. { + \mathbb{E}[{\rm{max}}({\rm{0,1}} + {D_k}(x,\hat y))]} \right] \cr} $$

where * K=3* denotes the number of discriminator scales, and $\:{D}_{k}(x,y)$and $\:{D}_{k}(x,\widehat{y})$ represent the responses of the * k*-th scale PatchDiscriminator to real (H&E, IHC) pairs and generated (H&E, $\:\widehat{y}$) pairs, respectively, conditioned on the score label. This objective penalizes the misclassification of real samples as fake and generated samples as real, while the Hinge margin of ± 1 provides a bounded, non-vanishing gradient signal that mitigates discriminator saturation commonly observed with standard binary cross-entropy losses.

#### Loss weight schedule

The scalar weighting coefficients $$\:\left\{{\lambda\:}_{i}\right\}$$ follow a two-phase annealing schedule designed to prioritize stable structural reconstruction during early training before progressively emphasizing perceptual sharpening and adversarial realism. During the warm-up phase (epochs 1–15), reconstruction terms are emphasized, $$\:{\lambda\:}_{L1}\:=\:8.0,\:\:{\lambda\:}_{FM}\:=\:15.0,\:{\:\lambda\:}_{SSIM}=\:0.6\:$$, to establish structural correspondence and prevent early adversarial collapse before the discriminator has reached sufficient discriminative capacity. After epoch 15, reconstruction weights are relaxed—$$\:{\lambda\:}_{L1}\:=\:5.0,\:\:{\lambda\:}_{FM}=\:10.0,\:\:{\:\lambda\:}_{SSIM}\:=\:0.35$$, allowing the perceptual and adversarial terms to sharpen chromogenic texture and membrane detail. The following coefficients remain fixed throughout both training phases: $$\:{\lambda\:}_{adv}\:=\:1.0,\:\:{\lambda\:}_{LPIPS}\:=\:12.0,\:\:{\lambda\:}_{edge}=\:4.0,$$$$\:{\lambda\:}_{DAB1}=\:5.0,\:\:{\lambda\:}_{H1}=\:1.0,$$$$\:\:{\lambda\:}_{DAB2}\:=\:10.0,\:\:{\lambda\:}_{ds}\:=\:2.0,$$$$\:{\lambda\:}_{color}\:=\:3.5,\:\:{\lambda\:}_{contr}\:=\:0.015,\:\:{\lambda\:}_{var}=\:0.007$$. The learning rate schedule for both generators initiates with a 5-epoch linear warm-up from zero to the nominal value, followed by a constant phase for the remainder of the 50-epoch training run.

The loss weights were determined through an iterative validation-driven tuning process. Coefficients were adjusted across experimental configurations in response to observed validation metrics—primarily FID, LPIPS, and SSIM—with multiple terms tuned simultaneously between experimental configurations when their effects were interdependent, such as the balance between $\:{\lambda\:}_{L1\:}$and $\:{\lambda\:}_{FM}$ during the transition between training phases. The final two-phase schedule was selected after repeated empirical refinement. During the early training stage, higher reconstruction weights were used to establish stable structural correspondence before the discriminator became sufficiently reliable. After generator loss stabilized, the reconstruction weights ($\:{\lambda\:}_{L1}$, $\:{\lambda\:}_{FM}$, and $\:{\lambda\:}_{SSIM}$) were reduced, allowing the adversarial and perceptual objectives to emphasize high-frequency texture refinement without sacrificing structural consistency. The remaining loss coefficients were kept fixed throughout training once a stable validation performance between reconstruction fidelity, perceptual realism, and optimization stability had been achieved.

### Training and inference pipeline

The complete pipeline, illustrated in Fig. 1, comprises a training phase with iterative optimization and an inference phase with deterministic feedforward synthesis. **Training** proceeds sequentially through the following stages:

1. **Data Preparation and Normalization**: H&E/IHC image pairs are resized to $\:512\times\:512$, normalized to $\:\left[-\mathrm{1,1}\right]$, and augmented with random rotations, flips, color jitter, and additive noise. A **Weighted Random Sampler** assigns sampling weights inversely proportional to class frequency, ensuring balanced representation of all four HER2 scores (0, 1+, 2+, 3+) across batches without discarding minority-class samples.
2. **Stage 1 — Stain Mapping (**$\:{\boldsymbol{G}}_{1}$**)**: The H&E image is downsampled to $\:256\times\:256$ and processed by $\:{G}_{1}$ to predict a two-channel stain prior (Hematoxylin and DAB).
3. **Input Fusion**: The full-resolution H&E image and the upsampled stain map are concatenated to form the five-channel input tensor for $\:{G}_{2}$. The target HER2 score is embedded and injected at multiple scales within $\:{G}_{2}$.
4. **Stage 2 — Refined Generation (**$\:{\boldsymbol{G}}_{2}$**)**: The refinement generator synthesizes the full-resolution IHC image, with an auxiliary branch at the first decoder upsampling stage providing intermediate supervision.
5. **Multi-Scale Discrimination (**$\:\boldsymbol{D}$**)**: The discriminator evaluates real and generated IHC images at three spatial resolutions, producing patch-level confidence maps and intermediate features for the generator’s feature-matching objective.
6. **Optimization and Model Selection**: Generator and discriminator parameters are updated via the 13-term hybrid loss. Checkpoints are selected according to a composite validation score of FID, LPIPS, and SSIM.
7. **EMA Stabilization**: An exponential moving average of the $\:{G}_{2}$ weights is maintained with momentum increasing from 0.99 to 0.999 throughout training. The EMA model is used for validation and deployed at inference.

**Inference**: At deployment, the pipeline executes a deterministic two-stage forward pass: the normalized H&E image is processed sequentially by the $\:{\:G}_{1}$and the EMA-stabilized $\:{G}_{2}$. The output is denormalized from $\:[-1,\:1]$ to $\:[0,\:1]$ to produce the final synthesized IHC image.

## Experiments and results

This section presents a comprehensive empirical evaluation of the proposed two-stage generative framework across two benchmark datasets, BCI and HER2match, covering dataset descriptions, implementation details, baseline comparisons, and evaluation metrics. Quantitative and qualitative results are reported to assess the framework’s performance in terms of image fidelity, perceptual quality, distributional realism, and score-conditional synthesis fidelity.

### Datasets

The proposed framework is evaluated on two publicly available benchmarks that differ in acquisition protocol and alignment precision, enabling assessment across both adjacent-section and same-slide conditions. This subsection describes each dataset’s composition, resolution, and split strategy, followed by a discussion of the key technical challenges they present.

#### BCI dataset

**Fig. 3:**
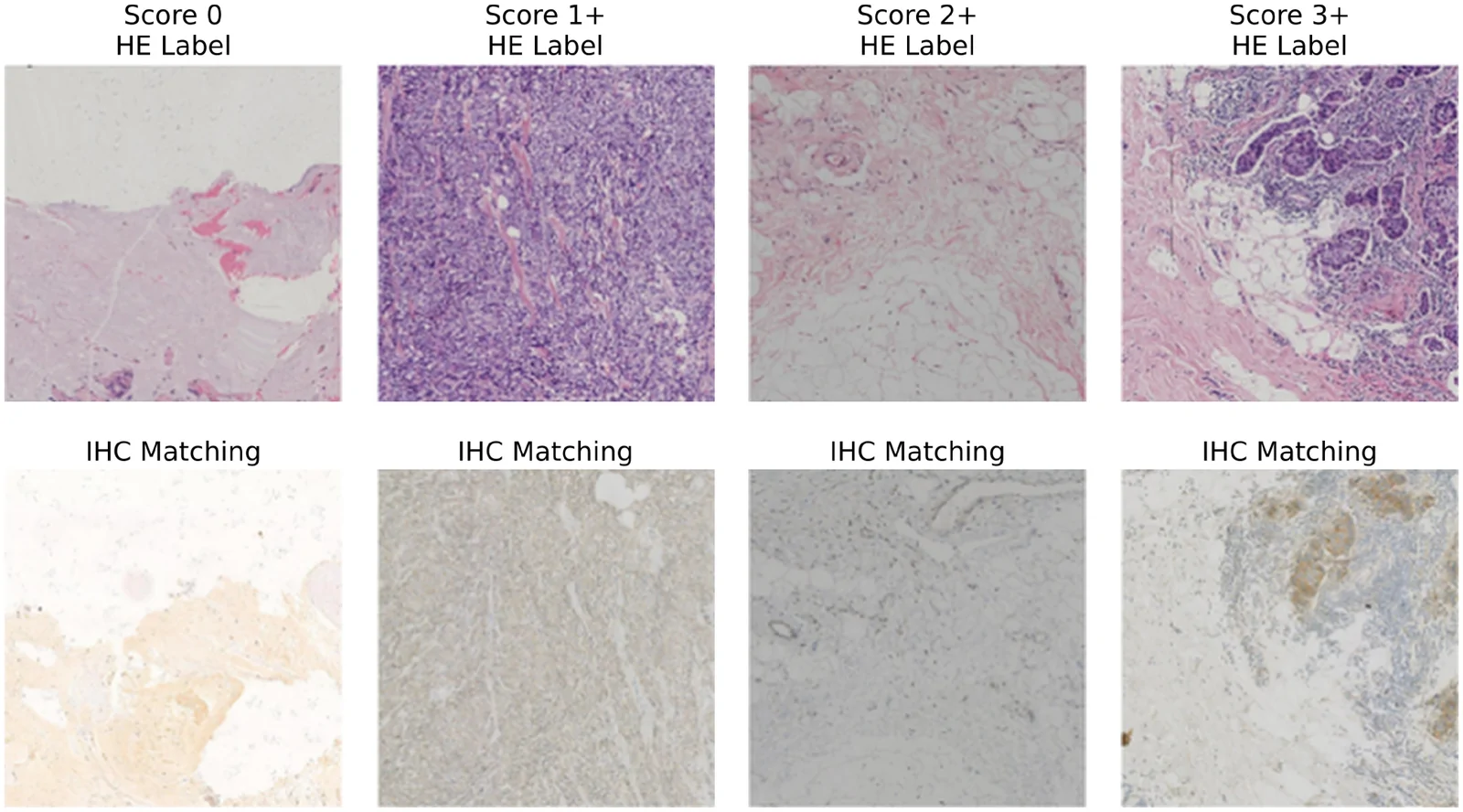
Representative H&E (top) and corresponding HER2-IHC (bottom) image pairs from the BCI dataset, illustrating the full spectrum of HER2 expression scores: 0 (negative), 1+ (weak/incomplete), 2+ (moderate), and 3+ (strong circumferential)

The proposed framework is evaluated on the Breast Cancer IHC (BCI) dataset [14], a large-scale benchmark curated specifically for H&E-to-HER2 virtual staining. Images were acquired using a Hamamatsu NanoZoomer S60 scanner at 0.46 μm/pixel. To address spatial misalignments inherent to consecutive tissue sections, a dual-stage registration protocol was applied, comprising coarse manual projection followed by fine-grained non-rigid registration using the Elastix toolbox. The curated dataset comprises 4,870 high-resolution image pairs (1024 × 1024 pixels), filtered to remove sectioning artifacts and blank regions. As shown in Fig. 3, the patches encompass the full clinical spectrum of HER2 expression from Score 0 to Score 3+, providing a robust foundation for conditional synthesis training and evaluation.

#### HER2match dataset

**Fig. 4:**
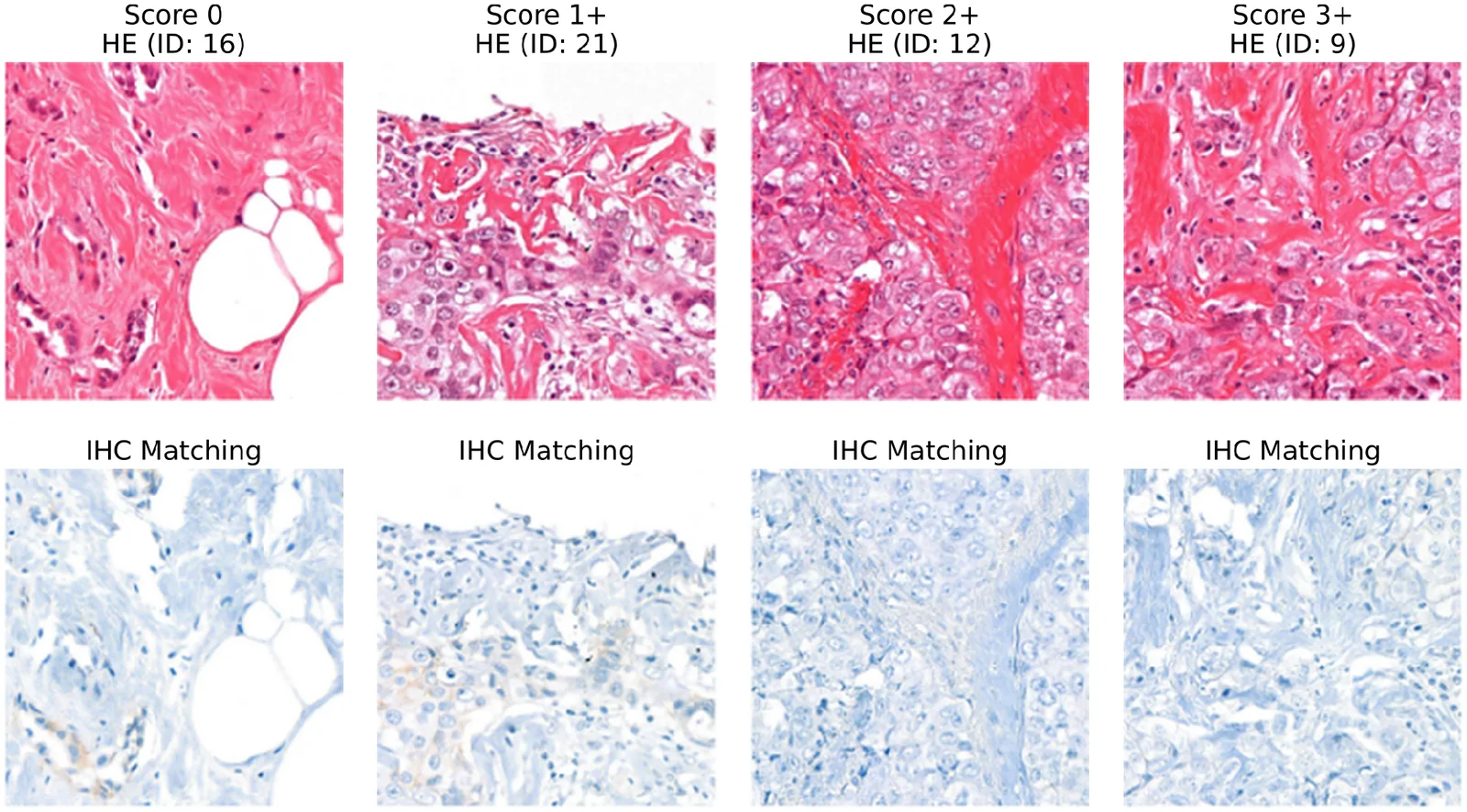
Representative H&E/IHC image pairs from the HER2match dataset across all four HER2 expression scores (0, 1+, 2+, 3+). The same-slide re-staining protocol ensures pixel-level spatial correspondence, eliminating adjacent-slice registration artifacts

To further validate framework robustness, experiments are additionally conducted on the HER2match dataset [9], which provides a high-precision same-slide re-staining benchmark. Unlike adjacent-slice datasets, HER2match employs a same-slide protocol on 17 whole-slide images (WSIs) of breast tissue: the tissue is initially stained with H&E and digitized, then chemically de-stained and re-stained for HER2 IHC. This methodology ensures that image pairs represent the identical physical tissue section, effectively eliminating biological variance and spatial misalignment from sequential sectioning. All tiles are provided at 0.25 μm/pixel resolution with standard 1024 × 1024-pixel dimensions. A per-patient-level split was implemented to prevent data leakage across training, validation, and testing subsets, as illustrated in Fig. 4.

#### Dataset challenges

**Fig. 5:**
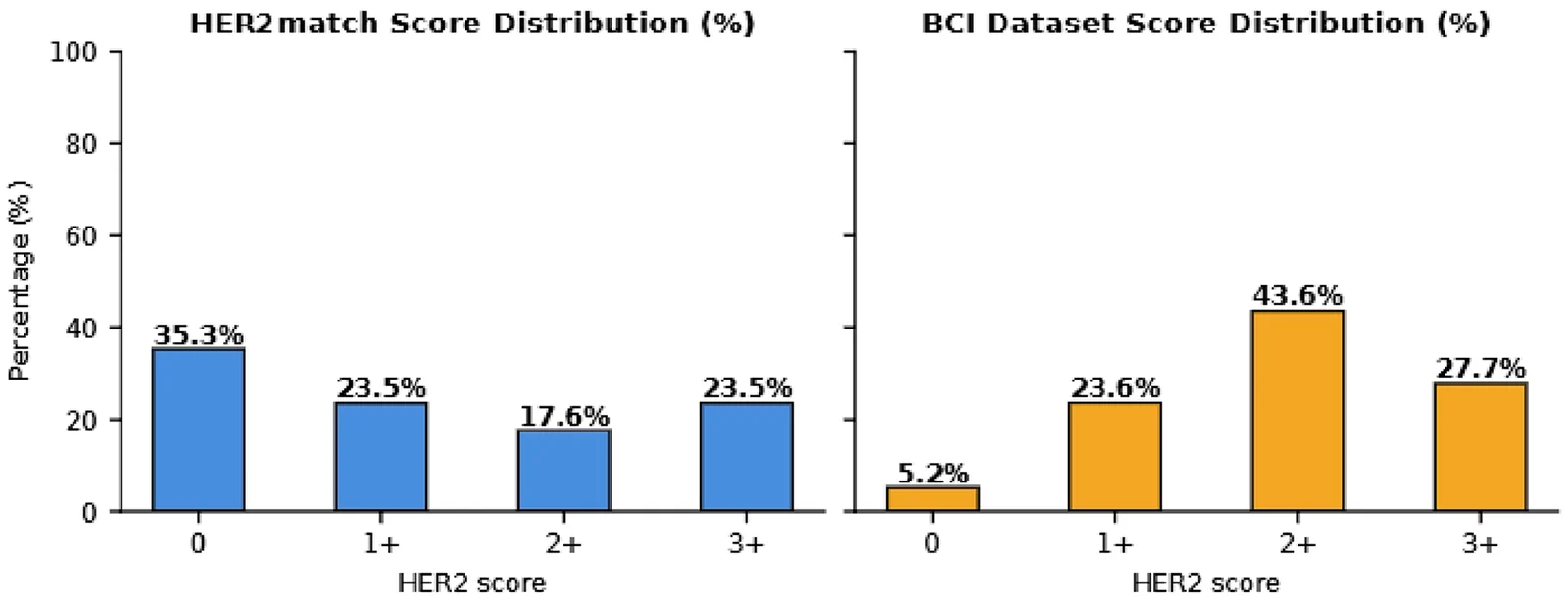
HER2 score distribution across the BCI (right) and HER2match (left) datasets

Both datasets present two primary technical challenges that informed the architectural design decisions of the proposed framework. First, **class imbalance**: the natural clinical distribution of HER2 scores is skewed (Fig. 5), as the BCI dataset is dominated by intermediate and high-expression HER2 scores, whereas HER2match exhibits a comparatively more balanced distribution. This is addressed through the Weighted Random Sampler described in Section “Training and inference pipeline”.

**Fig. 6:**
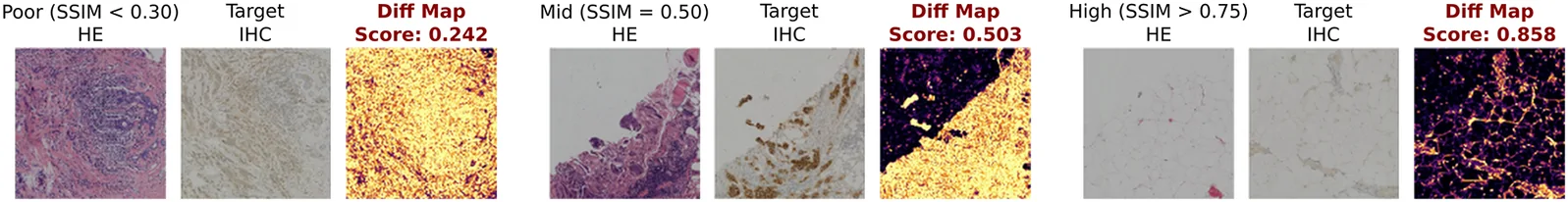
Illustration of spatial misalignment between paired H&E and IHC tiles in the BCI dataset, where intensity in the difference maps is proportional to local structural discrepancy, with high-intensity (yellow) regions reflecting severe misalignment and low-intensity (dark) regions indicating well-aligned tissue structures

Second, **spatial misalignment**: significant structural variance exists between H&E and IHC pairs in the BCI dataset, arising from adjacent tissue sectioning and evident in Elastix registration overlays, as visualized in Fig. 6. These local structural discrepancies motivate the inclusion of the Feature Alignment Block (FAB) and the associated contrastive objective in G2, which encourage representation consistency despite imperfect spatial correspondence.

### Implementation details

The framework comprises a U-Net stain mapper $\:{\:G}_{1}$and a conditional encoder–decoder refinement generator $\:G_2$ with a nine-block ResNet bottleneck, trained alongside a three-scale PatchGAN discriminator D for 50 epochs. All experiments use $\:512\:\times\:512$ input resolution with a batch size of 6. Optimization employs AdamW with a two-time-scale update rule (TTUR): the generator encompassing both $\:{\:G}_{1}\:$and $\:G_2$ uses a learning rate of $\:1\times\:{10}^{-4}$, while the discriminator uses $\:4\times\:{10}^{-4}$. The learning rate schedule begins with a 5-epoch linear warm-up from zero, and holds constant throughout the remaining training epochs. Mixed-precision training with automatic gradient scaling is used throughout. An exponential moving average of the $\:G_2\:$parameters is maintained with momentum increasing from 0.99 to 0.999 across training, and this EMA copy is used for validation and final inference. All experiments were conducted on Kaggle’s NVIDIA T4 GPU with 16 GB of memory.

### Performance evaluation metrics

Evaluation employs three complementary categories of metrics: pixel-level fidelity, perceptual similarity, and distributional alignment.

#### Classical structural fidelity metrics

Classical structural fidelity metrics, such as **PSNR** and **SSIM**, evaluate the quality of synthesized images by measuring pixel-level accuracy and perceptual similarity to ground-truth references. These metrics quantify pixel-level reconstruction accuracy and local structural correspondence, which serve as necessary but not sufficient conditions for pathological fidelity.

##### **Peak Signal-to-Noise Ratio (PSNR)**

[35] quantifies reconstruction accuracy as the logarithmic ratio of the maximum representable signal power to the mean squared reconstruction error, providing a pixel-level measure of synthesis artifact suppression.

22 $$\:PSNR=10\cdot\:{{log}}_{10}\left(\frac{{1.0}^{2}}{MSE}\right)$$

where $$\>MSE = {1 \over {C \times \>H \times \>W}}\sum \> _{c = 1}^C\sum \> _{i = 1}^H$$$$\sum \> _{j = 1}^W{\left[ {y\left( {c,i,j} \right) - \hat y\left( {c,i,j} \right)} \right]^2}$$ is the per-pixel Mean Squared Error between the ground-truth IHC image *y* and the synthesized image *\hat y*, averaged over all color channels *c* and spatial positions $$\:(h,\:w)$$. Higher PSNR values indicate closer pixel-level correspondence and fewer synthesis artifacts.

##### **Structural Similarity Index Measure (SSIM)**

[12] is a perception-based image quality metric that decomposes the similarity between two images into three independent structural components, luminance, contrast, and spatial structure, each of which is directly relevant to the preservation of cellular morphology, nuclear detail, and membrane boundary integrity in pathological images.

23 $$\:SSIM\left(x,y\right)=\frac{\left(2{\mu\:}_{x}{\mu\:}_{y}+{c}_{1}\left)\right(2{\sigma\:}_{xy}+{c}_{2}\right)}{\left({\mu\:}_{x}^{2}+{\mu\:}_{y}^{2}+{c}_{1}\left)\right({\sigma\:}_{x}^{2}+{\sigma\:}_{y}^{2}+{c}_{2}\right)}$$

where $\:{\mu\:}_{x}$ and $\:{\mu\:}_{y}$ are the local mean luminances of the generated and reference images, respectively; $\:{\sigma\:}_{x}^{2}$ and $\:{\sigma\:}_{y}^{2}$ are their local variances; $\:{\sigma\:}_{xy}$ is the local cross-covariance; and $\:c_1\:=\:(k_1L)^2$, $\:c_2\:=\:(k_2L)^2$ are stabilizing constants with * L* denoting the pixel value dynamic range and $\:k_1\:=\:0.01$, $\:k_2\:=\:0.03$. $\:SSIM\:\in\:\:[0,\:1]$, with a value of 1 indicating perfect structural identity. SSIM is computed over 11 × 11 Gaussian-weighted local windows and averaged across the full image.

#### Perceptual and distributional realism metrics

Perceptual and distributional realism metrics, including **FID**, **LPIPS**, and **KID**, assess generative model performance by comparing deep feature distributions and human-aligned perceptual similarities between synthesized and real IHC images. These advanced measures outperform pixel-based metrics like PSNR and SSIM in capturing perceptual plausibility, statistical alignment, and visual fidelity in histopathological virtual staining.

**Fréchet Inception Distance (FID)** [36] is the primary distributional realism metric for evaluating generative model quality. FID measures the Fréchet (Wasserstein-2) distance between two multivariate Gaussian distributions fitted to Inception-v3 deep feature representations of the real (r) and generated (g) image sets:

24 $$\:FID=\parallel\:{\mu\:}_{r}-{\mu\:}_{g}{\parallel\:}^{2}+Tr({{\Sigma\:}}_{r}+{{\Sigma\:}}_{g}-2({{\Sigma\:}}_{r}{{\Sigma\:}}_{g}{)}^{1/2})$$

where $\:{\mu\:}_{r}$, $\:{\mu\:}_{g}$ are the mean Inception feature vectors of the real and generated image sets, respectively; $\:{{\Sigma\:}}_{r},\:{{\Sigma\:}}_{g}$ are the corresponding covariance matrices; $\>Tr({\rm{\cdot}})$ denotes the matrix trace; and $\:({{\Sigma\:}}_{r}{{\Sigma\:}}_{g}{)}^{1/2}$ is the matrix square root of the product covariance. FID = 0 if and only if the two distributions are identical. Lower FID values indicate stronger statistical alignment between the synthesized IHC distribution and the real benchmark IHC distribution [9, 20].

##### **Learned Perceptual Image Patch Similarity (LPIPS)**

[33] measures perceptual distance in the deep feature space of a pre-trained VGG-16 network. For a generated image x and reference x₀

25 $$\:d(x,{x}_{0})={\sum\:}_{l}\frac{1}{{H}_{l}{W}_{l}}{\sum\:}_{h,w}\parallel\:{w}_{l}\odot\:({\widehat{y}}_{hw}^{l}-{\widehat{y}}_{hw}^{l,0}){\parallel\:}_{2}^{2}$$

where $\:{\widehat{y}}_{hw}^{l}\:$and $\:\:{\widehat{y}}_{hw}^{l,0}\:$are the unit-normalized deep feature activations at spatial location $\:(\mathrm{h}\:,\mathrm{w})\:$extracted from the * l*-th VGG-16 layer of the generated and reference images, respectively; $\:{w}_{l}$denotes the learned channel-wise importance weights; $\:{H}_{l}\:$and $\:{W}_{l}\:$are the spatial dimensions of the corresponding feature map; and ⊙ denotes element-wise multiplication. Lower LPIPS values indicate stronger perceptual similarity between synthesized and reference images.

##### **Kernel Inception Distance (KID)**

[37] provides a statistically unbiased alternative to FID by measuring the squared Maximum Mean Discrepancy (MMD) between Inception-v3 feature distributions of the real and generated image sets, using a polynomial kernel $\>k({\rm{\cdot}},\>{\rm{\cdot}})$ of degree $\:d\:=\:3$

26 $$\eqalign{& \>KID({P_r},{P_g}) = {\mathbb{E}_{x,{x^{\prime {\kern 1pt} }} \sim \>{P_r},y,{y^{\prime {\kern 1pt} }} \sim {P_g}}} \cr & [k(x,{x^{\prime \>}}) + k(y,{y^{\prime \>}}) - 2k(x,y)] \cr} $$

where $\:{P}_{r}$ and $\:{P}_{g}$ denote the real and generated image distributions, respectively; x, x’ ~ $\:{P}_{r}$ and $\:y,{y}^{{\prime\:}}\sim$ $\:{P}_{g}$ are independent samples from each distribution; and $\:k\left(u,v\right)={\left(\frac{1}{d}{u}^{T}v+1\right)}^{3}$is the polynomial kernel function with degree d = 3. KID has zero bias in the sense that KID = 0 if and only if P*r* = P*g*, regardless of the sample size, in contrast to FID, which is positively biased for small evaluation sets. KID is therefore particularly appropriate for the HER2match benchmark, which comprises a smaller number of test images. KID is reported with its standard deviation across multiple evaluation subsets to quantify the statistical reliability of the estimated distributional distance. Lower KID values indicate stronger distributional similarity between synthesized and real IHC images.

### Quantitative results

Table 2 presents a comprehensive quantitative comparison of the proposed method against all baseline models on both the BCI and HER2match test splits. Evaluation metrics include PSNR (structural pixel fidelity), SSIM (perceptual structural similarity), LPIPS (deep feature perceptual distance), FID (feature distribution similarity to real IHC), and KID (unbiased distributional distance with statistical confidence intervals). Best results per metric per dataset are highlighted in **bold**.

**Table 2:** Quantitative comparison of the proposed framework against baseline models on BCI and HER2match test splits

Dataset	Method	PSNR↑	SSIM↑	LPIPS↓	FID↓	KID↓
BCI	Pix2pix	18.3770	0.4439	0.5529	139.87	0.1136 ± 0.0126
	PyramidPix2Pix	19.0575	**0.4808**	0.5331	106.27	0.0743 ± 0.0123
	ASP	12.2539	0.4443	0.5895	261.20	0.2168 ± 0.0244
	PSPStain	11.7732	0.4446	0.6032	297.00	0.3383 ± 0.0225
	Ours	**22.5032**	0.4773	**0.5279**	**59.67**	**0.0264 ± 0.0086**
HER2match	Pix2pix	17.8049	0.3445	0.5302	57.98	0.0404 ± 0.0124
	PyramidPix2Pix	16.5921	0.3504	0.5120	57.63	0.0446 ± 0.0135
	ASP	20.0921	**0.4541**	0.5108	121.84	0.1429 ± 0.0215
	PSPStain	17.4377	0.3352	0.5870	174.47	0.1996 ± 0.0251
	Ours	**20.6584**	0.4467	**0.4486**	**20.42**	**0.0124 ± 0.0092**

↑ indicates higher is better; ↓ indicates lower is better. Best results per metric per dataset are in bold

**Fig. 7:**
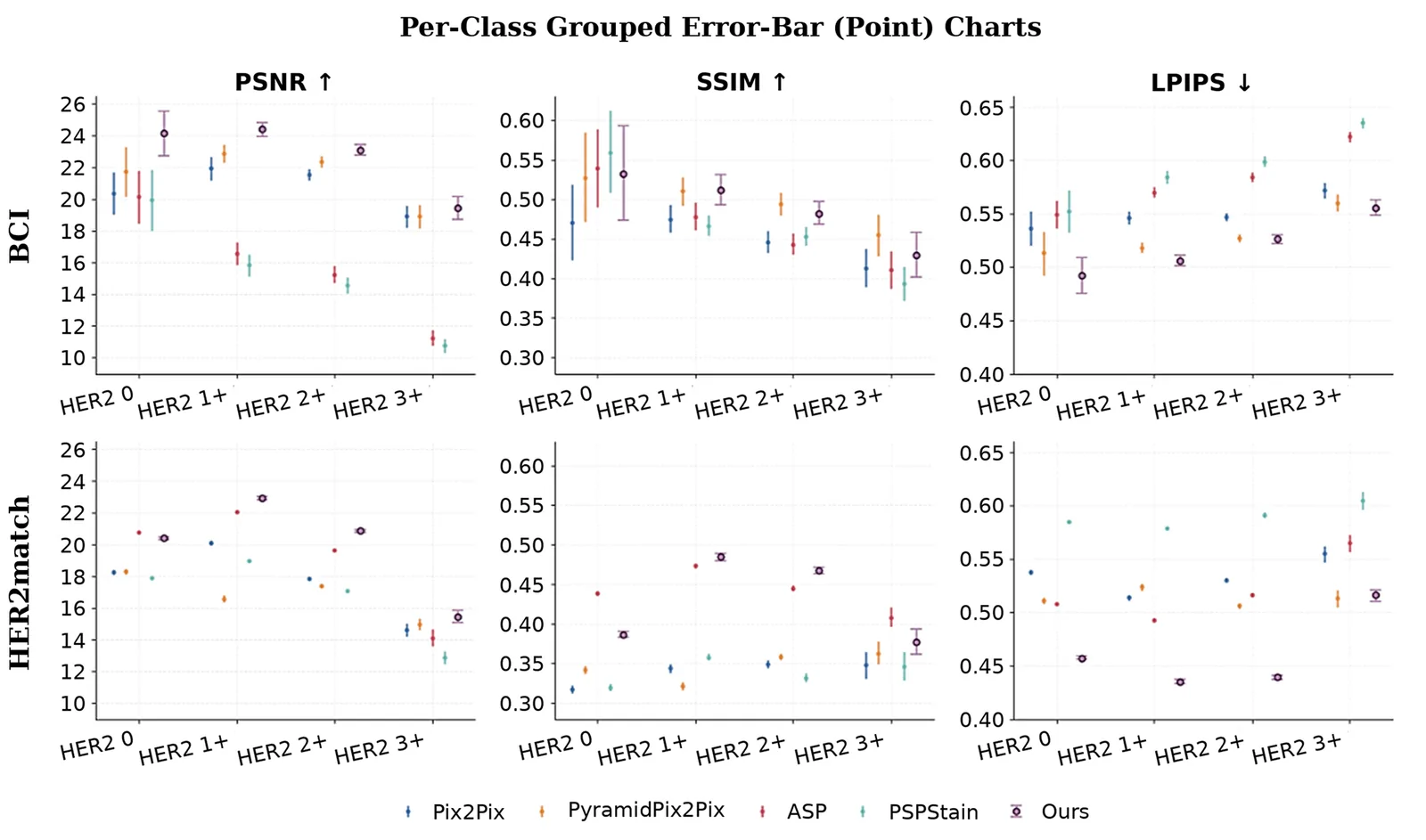
Per-class quantitative comparison of PSNR, SSIM, and LPIPS across the four HER2 scores on the BCI (top) and HER2match (bottom) datasets. Points denote the mean performance for each model, while error bars represent the 95% confidence intervals computed across the test samples of each score

**Fig. 8:**
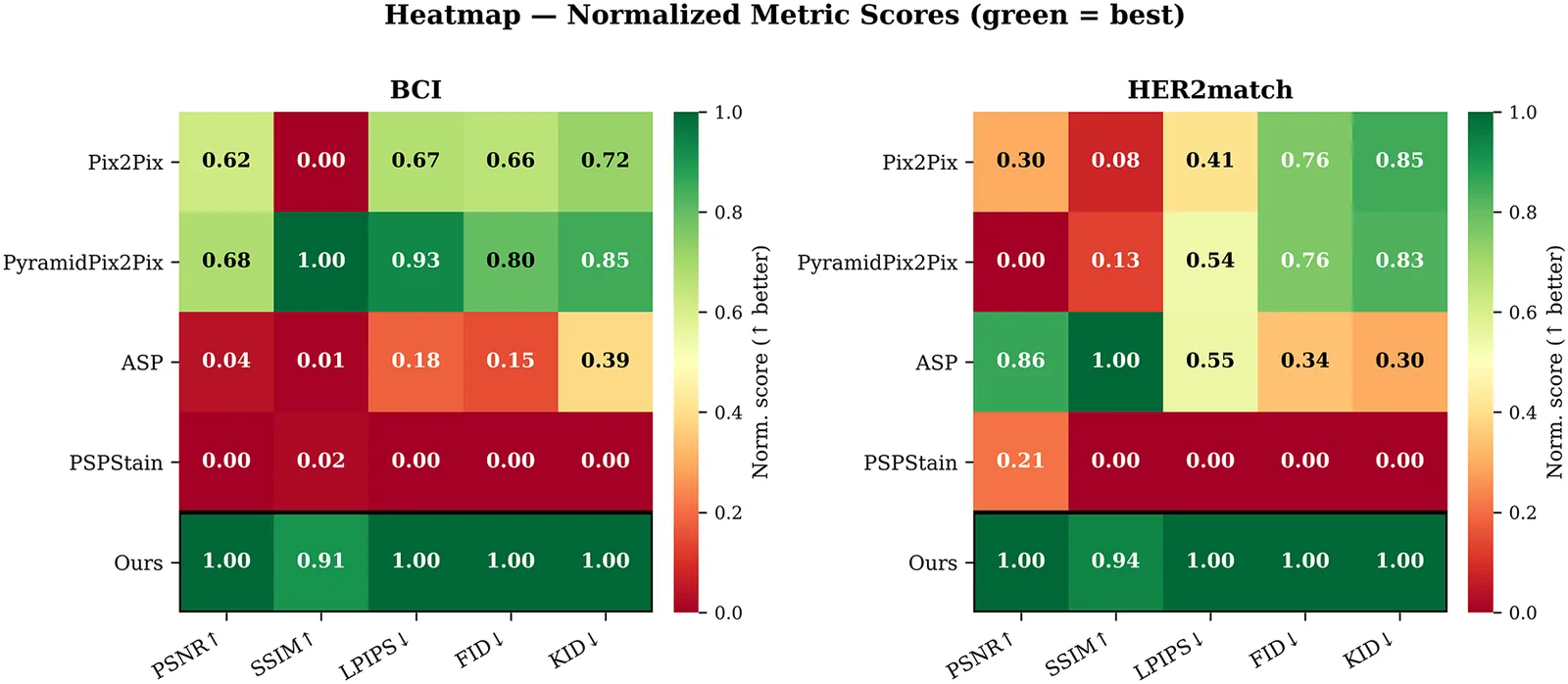
Heatmap of normalized metric scores for all models across BCI and HER2match datasets. Dark green cells indicate top-ranked performance per metric. The proposed method dominates four of five (PSNR, LPIPS, FID, KID) rankings on both datasets, confirming consistently superior distributional realism

**Fig. 9:**
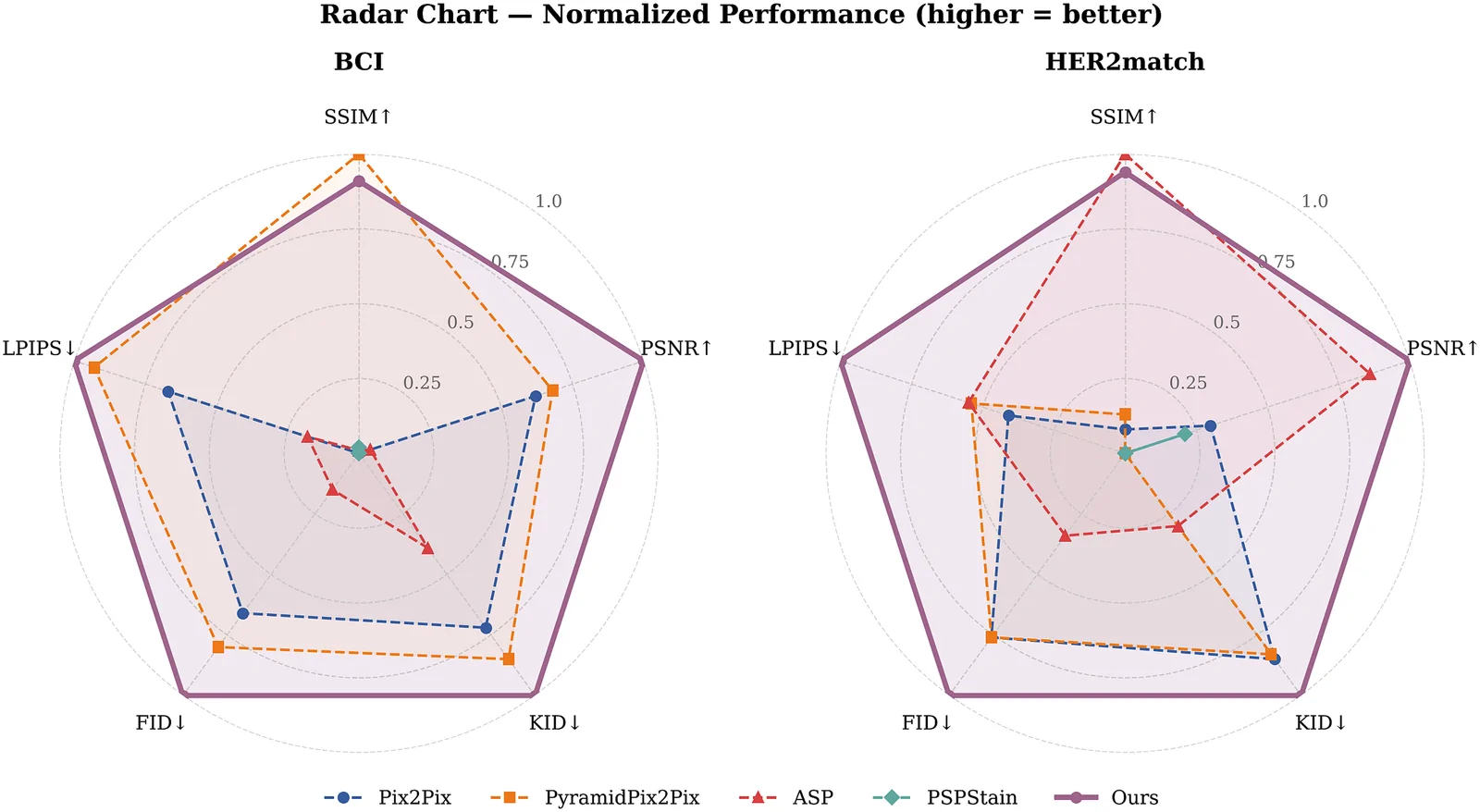
Normalized multi-metric radar charts for BCI (left) and HER2match (right), with lower-is-better metrics inverted so outward extent indicates superior performance. The proposed method occupies the outermost envelope on four of five axes on both datasets, prioritizing distributional and perceptual fidelity over pixel-wise structural matching (SSIM)

On the BCI dataset, the proposed method achieves the highest PSNR (22.50 dB), best LPIPS (0.5279), lowest FID (59.67), and lowest KID (0.0264 ± 0.0086), representing improvements of + 3.44 dB PSNR, − 46.60 FID, and − 64% KID over the strongest baseline (PyramidPix2pix). On the HER2match dataset, which provides same-slide re-stained pairs with substantially reduced sectioning misalignment and thus constitutes a more rigorous test of pure synthesis quality, the proposed method achieves the best PSNR (20.66 dB), LPIPS (0.4486), FID (20.42), and KID (0.0124 ± 0.0092), with an FID reduction of 64.6% over the nearest baseline (PyramidPix2pix). SSIM performance is competitive on both datasets; the marginal gap relative to PyramidPix2pix on BCI (0.4773 vs. 0.4808) is offset by substantial distributional gains, consistent with the known tendency of SSIM to favor locally-smooth outputs regardless of biological plausibility. The consistently superior FID and KID scores across both benchmarks confirm that the proposed framework generates IHC images whose feature distributions are closest to real benchmark IHC, which is the most stringent statistical criterion for distributional realism in virtual staining evaluation. Per-class breakdowns of PSNR, SSIM, and LPIPS across all four HER2 scores are presented in Fig. 7. Error bars denote 95% bootstrap confidence intervals, illustrating the statistical variability of each metric across the test samples. The proposed framework consistently maintains superior or competitive performance across all HER2 scores, with competitive performance maintained across all HER2 score categories, including the intermediate and high-expression ranges (2 + and 3+). A normalized metric heatmap across all models and both datasets is shown in Fig. 8, further corroborating the proposed method’s dominance on distributional metrics. Figure 9 presents radar charts that summarize the overall multi-metric profile of each model, highlighting the proposed framework’s balanced superiority across FID, KID, and LPIPS.

### Qualitative results

**Fig. 10:**
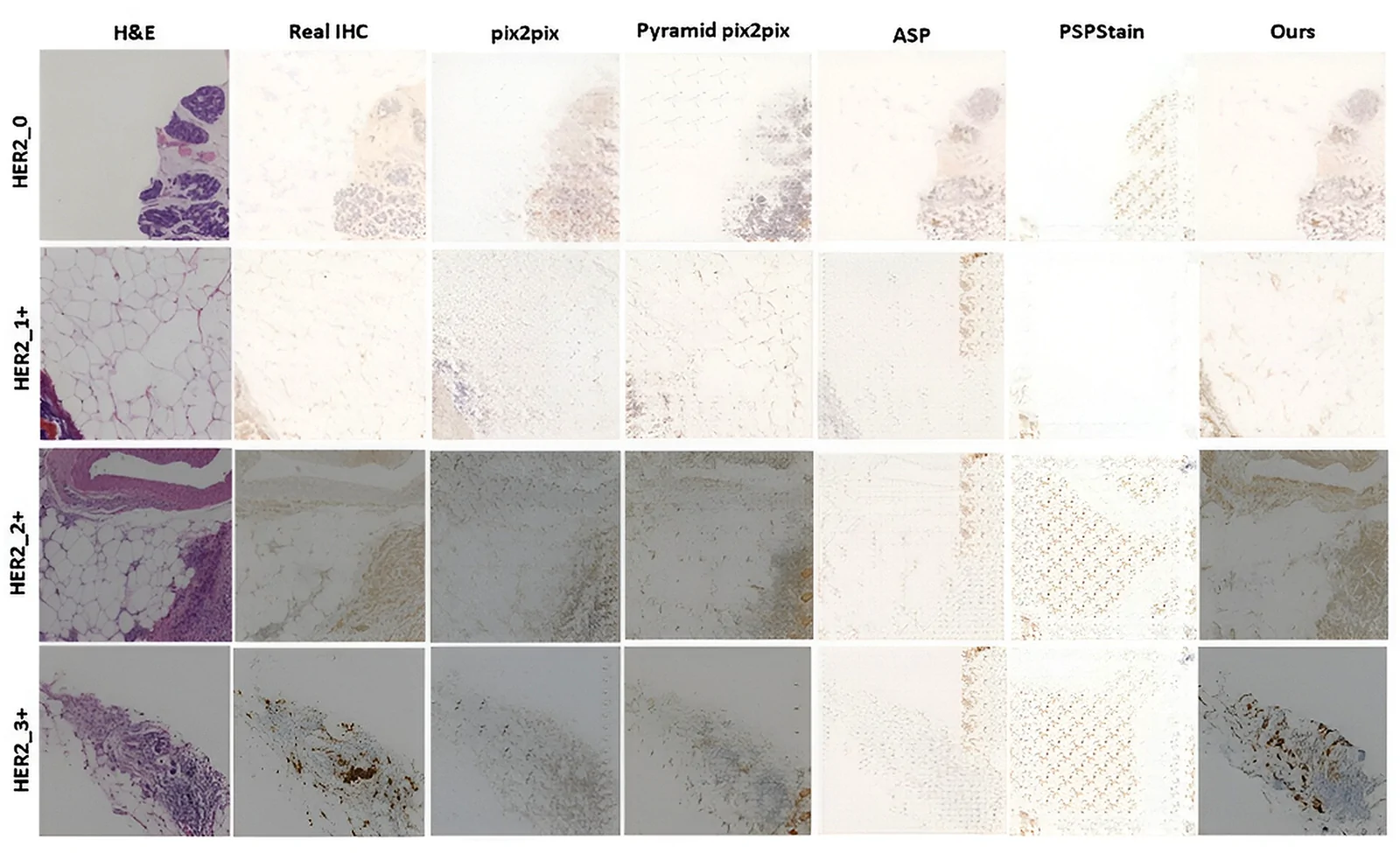
Qualitative comparison on representative BCI test samples across all four HER2 scores (0, 1+, 2+, and 3+). From left to right: H&E input, real IHC ground truth, Pix2pix, PyramidPix2pix, ASP, PSPStain, and the proposed method

**Fig. 11:**
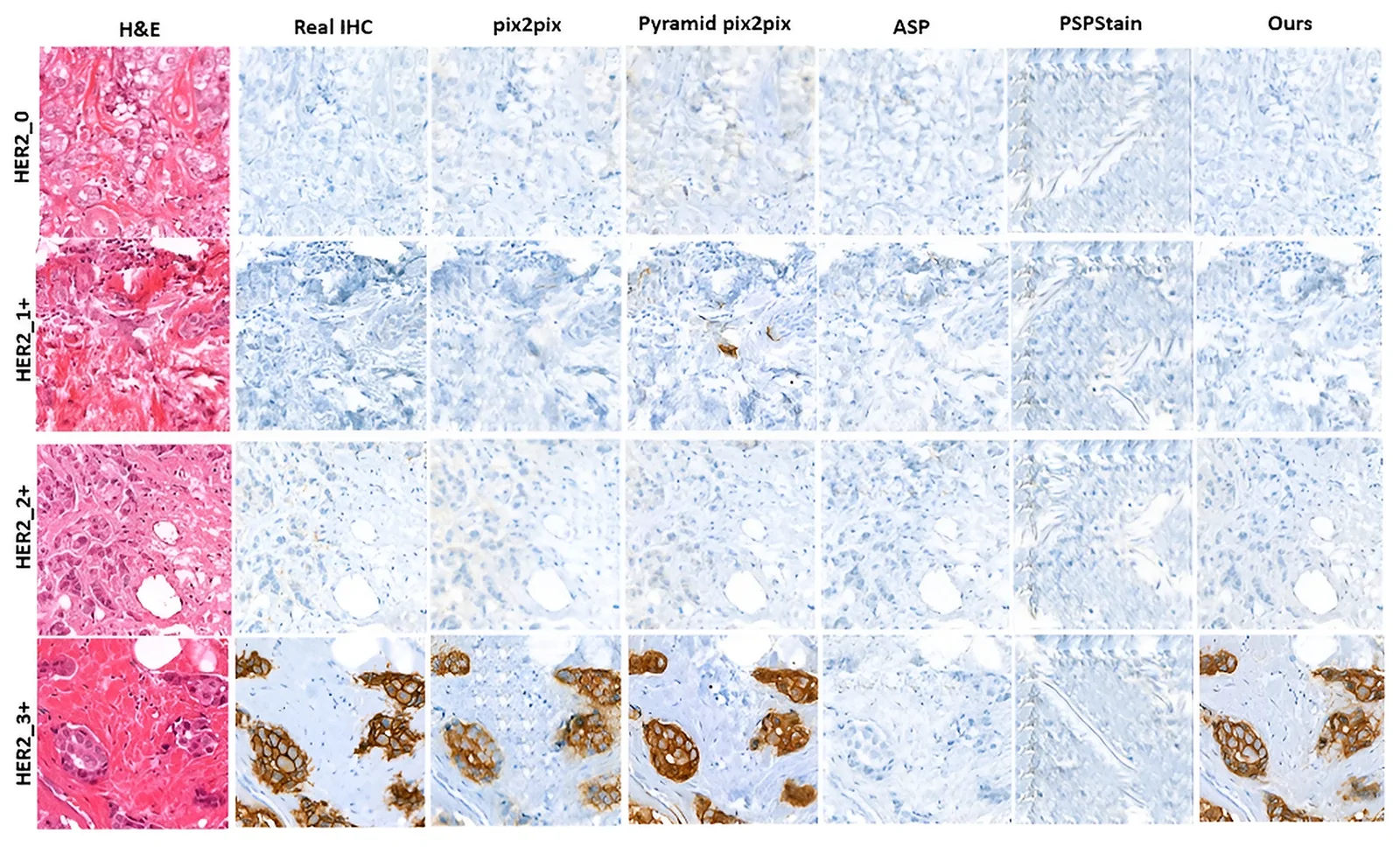
Qualitative comparison on representative HER2match test samples across all four HER2 scores (0, 1+, 2+, and 3+). From left to right: H&E input, real IHC ground truth, Pix2pix, PyramidPix2pix, ASP, PSPStain, and the proposed method

Figures 10 and 11 present qualitative comparisons of generated IHC images across all compared methods on representative BCI and HER2match test cases, respectively. The proposed method consistently produces IHC images with sharper membrane delineation, chromogenic distributions that more closely match the reference IHC images, and fewer hallucination artifacts compared to all baselines. In particular, boundary sharpness and structural edge coherence, which are associated with strong HER2 membrane staining patterns, are more reliably reproduced by the proposed framework, whereas baseline models exhibit incomplete staining patterns or spurious brown pigmentation in non-membrane regions.

### Comparison with diffusion-based models

To further position the proposed framework relative to recent generative approaches, we additionally compared it against two representative diffusion-based image translation models, **BBDM (Brownian Bridge Diffusion Model)** and **DDIB (Dual Diffusion Implicit Bridges)**, on both the BCI and HER2match datasets. For a consistent comparison, both baseline models were trained on identical data splits at the same resolution, using architecture-appropriate hyperparameters under comparable computational settings.

The DDIB model originally performs unpaired translation by composing two diffusion models trained independently on each domain. During inference, the source H&E image is encoded into a latent representation via the source domain’s probability-flow ODE, which the target domain’s reverse probability-flow ODE then decodes into the IHC image [38]. Conversely, BBDM establishes a direct mathematical “Brownian bridge” between the two domains. Instead of degrading the source image into pure noise, BBDM directly interpolates the HE image into the IHC target, learning to reverse this specific trajectory for inference [39].

Evaluation was performed using the same quantitative metrics (PSNR, SSIM, LPIPS, FID, and KID) and representative qualitative examples.

**Table 3:** Quantitative comparison of the proposed framework against diffusion-based models on BCI and HER2match test splits

Dataset	Method	PSNR↑	SSIM↑	LPIPS↓	FID↓	KID↓
BCI	BBDM	20.6353	**0.5962**	0.5985	122.97	0.1859 ± 0.0140
	DDIB	9.9043	0.0193	0.7728	351.75	0.4248 ± 0.0261
	Ours	**22.5032**	0.4773	**0.5279**	**59.67**	**0.0264 ± 0.0086**
HER2match	BBDM	19.9113	**0.4576**	0.5350	31.64	0.1458 ± 0.0184
	DDIB	6.4892	0.0159	0.7808	345.03	0.5686 ± 0.0559
	Ours	**20.6584**	0.4467	**0.4486**	**20.42**	**0.0124 ± 0.0092**

↑ indicates higher is better; ↓ indicates lower is better. Best results per metric per dataset are in bold

As summarized in Table 3, the proposed framework consistently outperforms both diffusion-based baselines across nearly all evaluation metrics on both datasets. On the BCI dataset, our method achieves the highest PSNR (22.50 dB), the lowest LPIPS (0.5279), the lowest FID (59.67), and the lowest KID (0.0264 ± 0.0086). Similar improvements are observed on HER2match, where our framework attains the best PSNR (20.66 dB), LPIPS (0.4486), FID (20.42), and KID (0.0124 ± 0.0092). Although BBDM achieves higher SSIM on both datasets, this advantage does not translate into improved perceptual or distributional realism, as reflected by its substantially higher FID and KID scores.

**Fig. 12:**
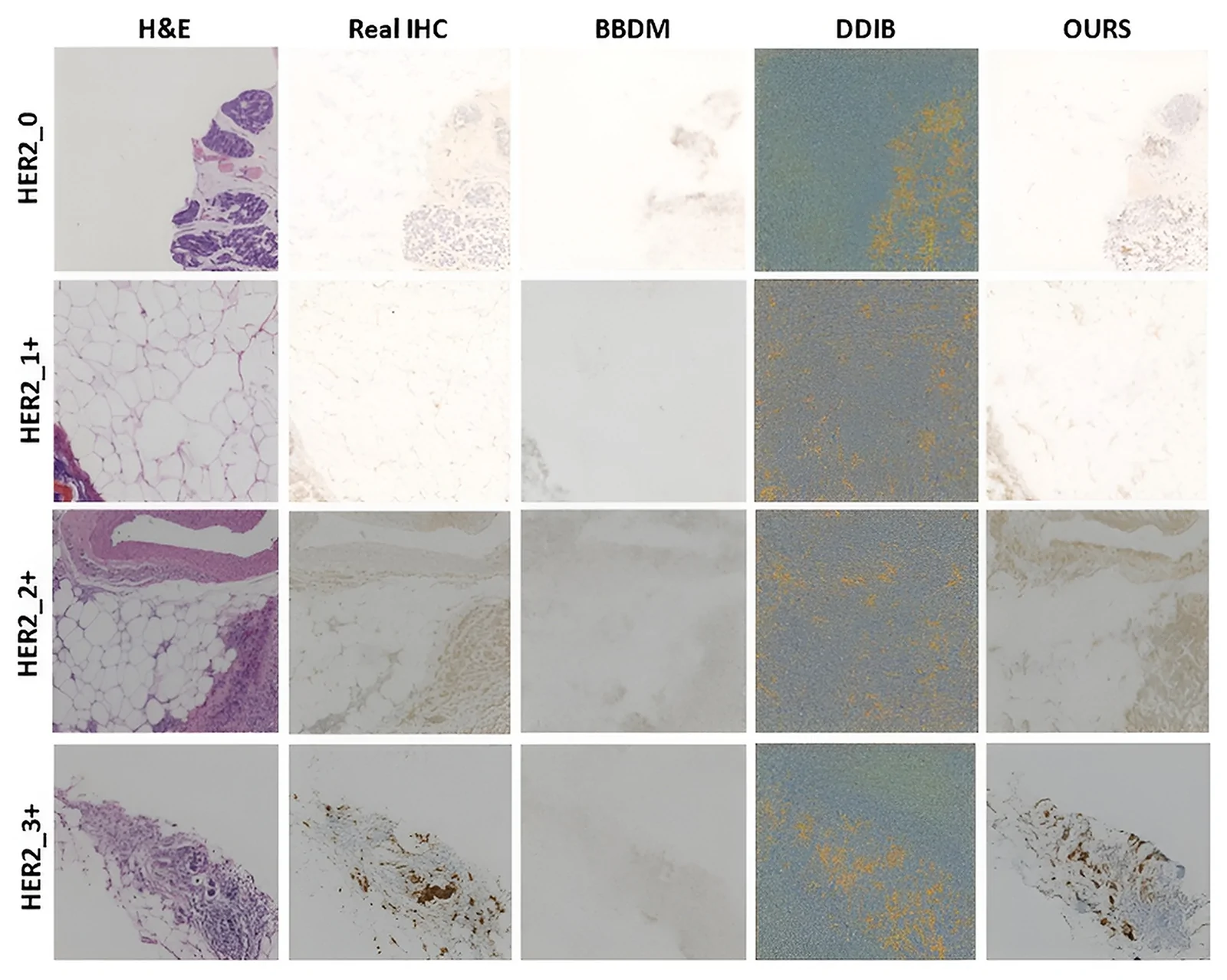
Qualitative comparison on representative BCI test samples across all four HER2 scores (0, 1+, 2+, and 3+). From left to right: H&E input, real IHC ground truth, BBDM, DDIB, and the proposed method

**Fig. 13:**
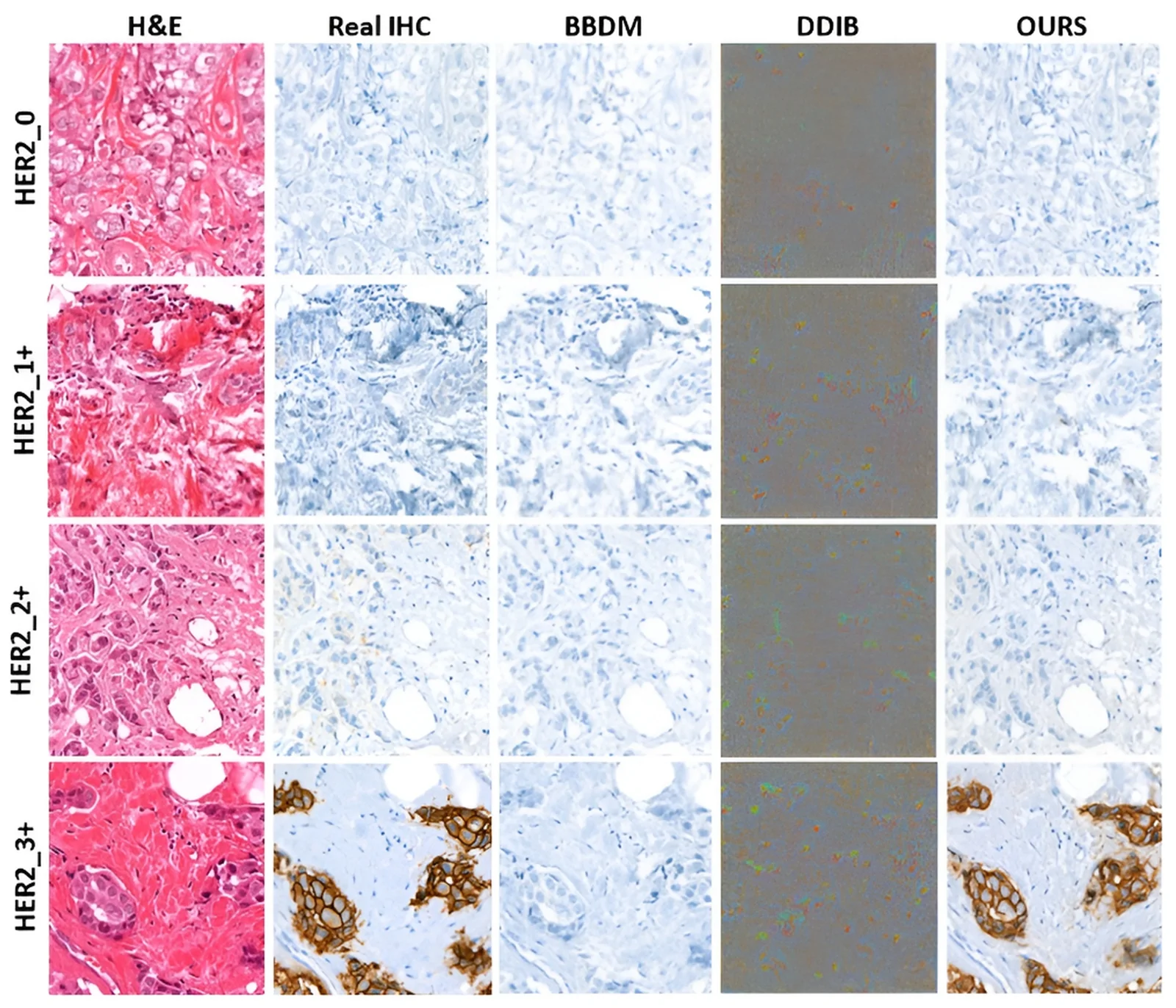
Qualitative comparison on representative HER2match test samples across all four HER2 scores (0, 1+, 2+, and 3+). From left to right: H&E input, real IHC ground truth, BBDM, DDIB, and the proposed method

The qualitative comparisons in Figs. 12 and 13 further support the quantitative findings. BBDM generally preserves coarse tissue appearance but often produces blurred membrane boundaries and weaker chromogenic contrast, particularly in HER2 2 + and 3 + samples. DDIB frequently introduces noticeable stochastic noise and fails to reconstruct staining patterns that visually correspond to the reference IHC images (Figs. 12 and 13). Consequently, the generated images exhibit reduced structural consistency, in agreement with the quantitative metrics reported in Table 3. Because DDIB relies on training two separate unconditional models, its overall training cost is computationally more expensive than that of the proposed framework. Under the identical 50-epoch training schedule adopted in this study, the proposed framework consistently produced superior image quality.

### Model explainability via integrated gradients attribution mapping (I2AM)

To interrogate the internal synthesis behavior of the proposed framework and verify that the two-stage architecture functions as designed—specifically, that the stain-prior channels predicted by $\:{G}_{1}$ are actively exploited by the refinement generator $\:{G}_{2}$ rather than ignored—we apply Integrated Gradients Attribution Mapping (I2AM) [40]. The method computes pixel-wise attribution scores by integrating gradients of a scalar target with respect to the 5-channel input tensor $\:{x}_{in}=\left[{I}_{HE};\widehat{S}\right]$ along a straight-line path from a baseline to the actual input:

27 $$\:I{G}_{j}\left(x\right)=\left({x}_{j}-{x}_{j}^{{\prime\:}}\right)\cdot\:{\int\:}_{0}^{1}\frac{\partial\:F\left({x}^{{\prime\:}}+{\upalpha\:}\left(x-{x}^{{\prime\:}}\right)\right)}{\partial\:{x}_{j}}d{\upalpha\:}$$

where $\:{x}^{{\prime\:}}$ is a constant-valued baseline tensor with all elements set to − 1—the lower bound of the normalized input range (representing a black reference image), and the scalar target $\:F(\cdot\:$) is defined as the squared L2 norm of the generated image:

28 $$\>F\left( x \right) = \left\| {{G_2}\left( {x,y} \right)} \right\|_2^2 = \sum \> _{c = 1}^3{\sum \> _{i,j}}\widehat {y_{c,i,j}^2}$$

**Fig. 14:**
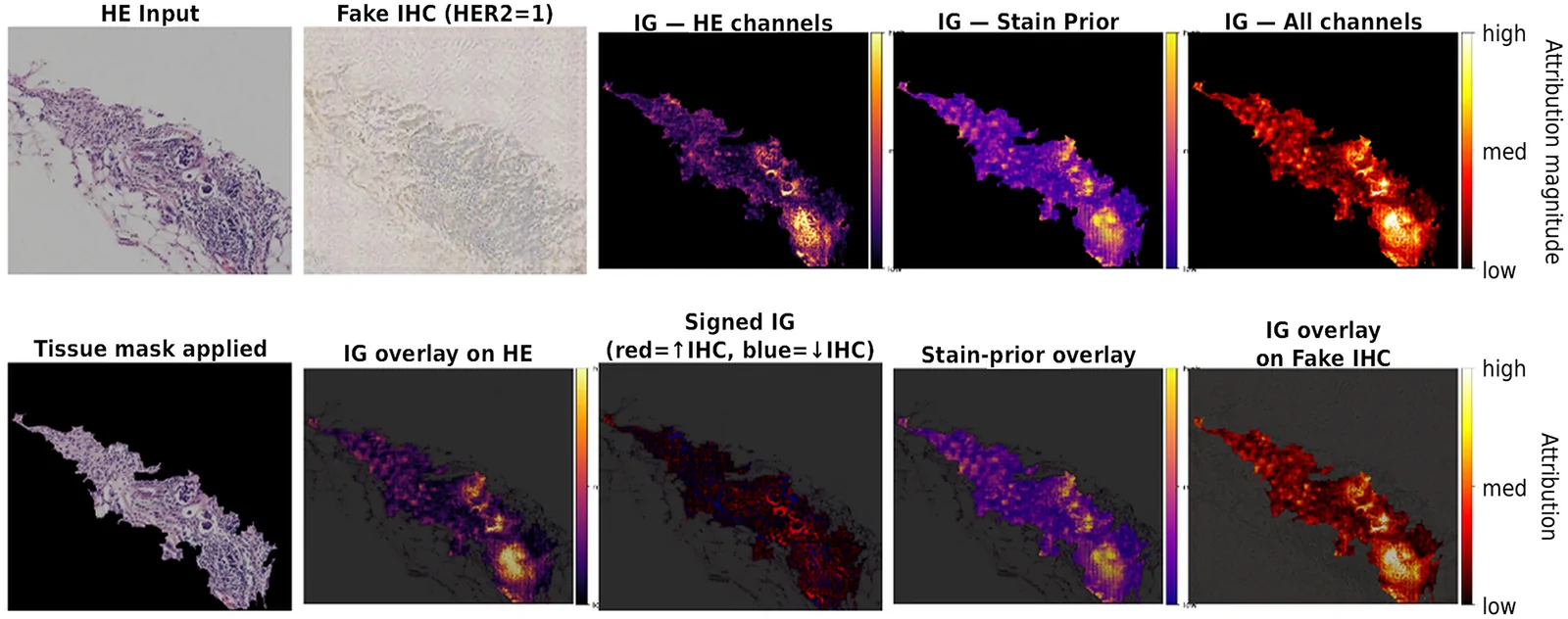
I2AM attribution panel for a BCI test image (HER2 = 1+, 50 steps, Otsu tissue mask). Top row: H&E input, generated IHC, and per-channel mean |IG| maps. Bottom row: Otsu-masked foreground, IG overlays, and signed attribution map. Per-channel mean |IG| values (StainCh1: 4.02, StainCh2: 2.99) confirm the dominance of stain-prior modalities over raw H&E channels

**Fig. 15:**
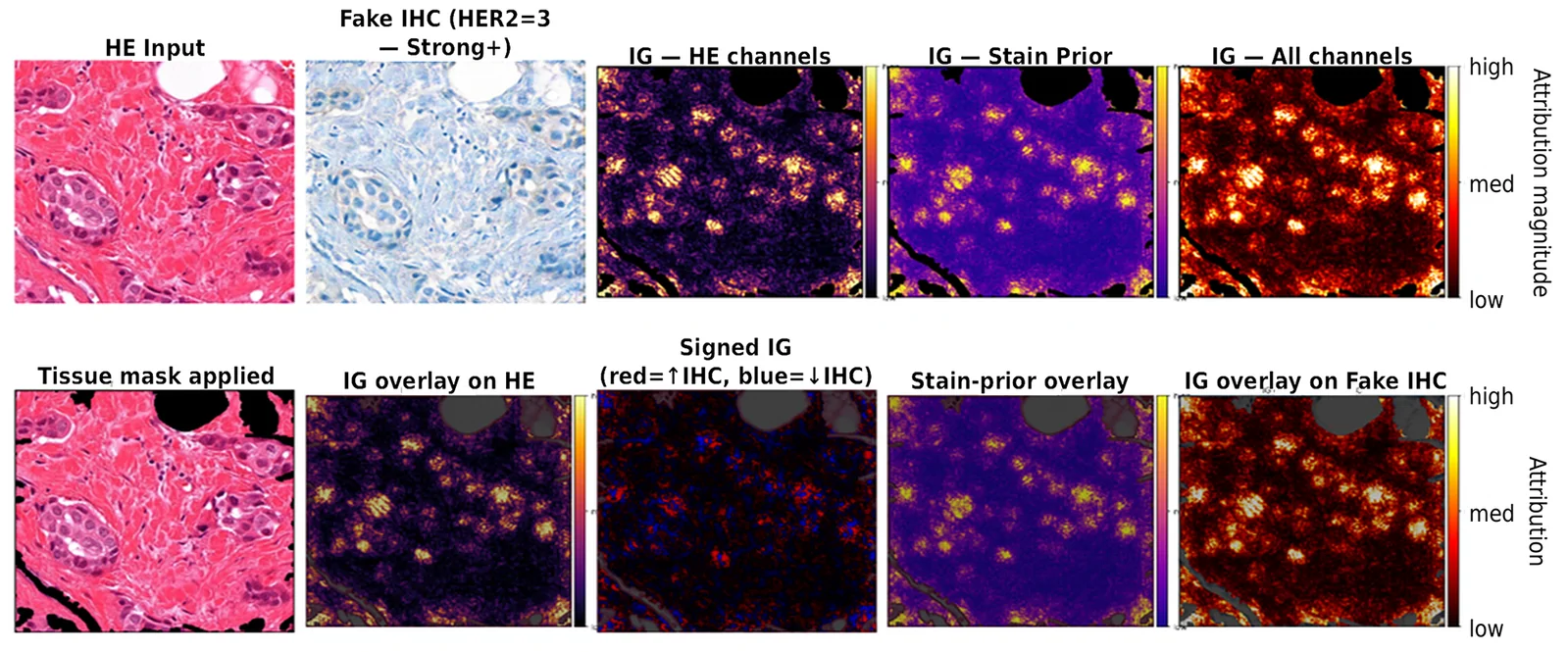
I2AM attribution panel for a HER2match test image (HER2 = 3+, 50 steps, Otsu tissue mask). Top row: H&E input, generated IHC, and per-channel mean |IG| maps. Bottom row: Otsu-masked foreground, IG overlays, and signed attribution map. The modality hierarchy shifts markedly (HE-R: 34.74 dominant), though stain-prior channels retain substantial attribution (~ 32% of total)

The squared output-energy objective was selected to capture global activation strength of the synthesized IHC response without restricting attribution to a specific spatial region or stain component. This target measures the overall intensity energy of the synthesized output. Attributions therefore identify which input channels and spatial regions most influence the overall output activation of the generator. The contribution of the predicted stain-prior channels is subsequently evaluated through the per-channel attribution analysis. The integral is approximated via a 50-step Riemann sum. Resulting maps are masked with an Otsu-thresholded tissue foreground mask to exclude background regions from quantitative analysis. In the signed IG visualizations (Figs. 14 and 15), red regions denote positive attribution—input features whose increase elevates the output energy (increasing the IHC staining intensity)—while blue regions denote negative attribution, where increasing the input feature suppresses output intensity.

Figure 14 presents the I2AM panel for a representative BCI test image conditioned on HER2 = 1+. The per-channel mean absolute attribution $\:\left|IG\right|\:$within the tissue mask reveals a clear modality hierarchy: StainCh1 (4.02) and StainCh2 (2.99) dominate over the individual H&E channels (HE-B: 2.85, HE-R: 2.46, HE-G: 1.75). This confirms that the refinement generator actively draws upon the stain-prior modality predicted by $\:{G}_{1}$, validating the architectural decision to inject explicit stain-chemistry information rather than relying solely on raw H&E morphology. Figure 15 shows the corresponding panel for a HER2match test image conditioned on HER2 = 3+. Here the modality hierarchy differs markedly: HE-R (34.74) is dominant, followed by StainCh1 (18.95), HE-B (17.93), HE-G (15.94), and StainCh2 (13.43). The stronger H&E signal reflects the higher native morphological quality and signal-to-noise ratio of the HER2match same-slide acquisition protocol, where H&E and IHC sections are same-slide aligned. Nevertheless, the stain-prior channels retain substantial absolute attribution, contributing approximately 32% of the total per-channel attribution compared to roughly 50% on BCI. While their relative contribution decreases on the higher-fidelity HER2match data, their continued functional role remains evident, supporting the effectiveness of the two-stage design even under optimal input conditions.

### Ablation study

To quantify the contribution of major architectural and optimization design decisions, we conduct a two-part ablation study on the BCI benchmark: a component-level ablation that evaluates each architectural group, and a loss-function ablation that isolates the effect of individual loss terms and the phased weight schedule. All configurations are evaluated on the full BCI validation set.

#### Component-level ablation

Table 4 reports a progressive component ablation; each configuration extends the previous one. Replacing the MSE adversarial objective with Hinge GAN loss (C1→C2) yields a 32.0% FID reduction relative to the C1 baseline, suggesting that the non-saturating gradient of Hinge loss provides a more effective training signal in this setting. Adding EMA smoothing on * G2*, a two-phase λ-schedule, and class-balanced sampling via WeightedRandomSampler (C3) reduces FID by a further 4.4% points, reaching − 36.4% relative to C1. Adding a Consistency-Aware Patch Loss (C4) degraded FID by + 18.4% relative to C3 and was therefore excluded from subsequent configurations.

Upgrading G2 to a nine-block ResNet bottleneck with a spectral-norm discriminator, 8-way augmentation, an image replay buffer, and learnable noise injection in the residual blocks (C5) produces a small regression relative to C3 (− 34.9% vs. −36.4% from C1, a difference of approximately 2 absolute FID points). The components introduced at C5 do not individually improve over C3; their benefit becomes apparent only after introducing the Multi-Scale Discriminator in C6, suggesting these components interact rather than contribute independently. C6 adds the Multi-Scale Discriminator with TTUR ($\:L{R}_{D}=4\times\:L{R}_{G}$), recovering to approximately the C3 FID level (− 35.7% vs. −36.4% from C1). Adding CBAM attention (C7) reduces FID by approximately 16 absolute points relative to C6 (− 47.9% from C1). The full model (C8) adds Sobel Edge Loss, yielding FID 59.67 — a 55.7% reduction from C1.

**Table 4:** Component-level additive ablation (BCI dataset)

Config	Description	ΔFID (%)
C1	Two-stage UNet + MSE GAN + HED stain losses	—
C2	C1 + Hinge GAN	−32.0
C3	C2 + EMA on G2 + Phased λ-schedule + Weighted Random Sampler	−36.4
C4	C3 + Consistency-Aware Patch Loss (**excluded** — degraded FID)	+ 18.4 (relative to C3)
C5	C3 + ResNet-9 bottleneck + Spectral-norm D + 8-way augmentation + Image replay buffer + Learnable noise injection	−34.9
C6	C5 + Multi-Scale Discriminator (Hinge, TTUR: LR_D = 4 × LR_G)	−35.7
C7	C6 + CBAM Attention	−47.9
C8 [Full Model]	**C7 + Sobel Edge Loss**	**−55.7**

ΔFID is relative to C1

#### Loss function ablation

Table 5 examines the influence of individual loss terms and the phased weight schedule on synthesis quality. Unlike the component-level ablation, these rows do not represent separately trained fixed-weight models; rather, they correspond to evaluation checkpoints sampled from a single continuous training run in which metrics were computed after every epoch. The first two rows therefore capture the model state immediately before and immediately after the scheduled phase transition.

The effect of the phase transition is clearly visible within the single training trajectory. At the end of Phase I (epoch 15), where $\:{\lambda\:}_{L1}=8.0$, $\:{\lambda\:}_{LPIPS}=12.0$, $\:{\lambda\:}_{FM}=15.0$, $\:{\lambda\:}_{SSIM}=0.6$, $\:{\lambda\:}_{DAB2}=10.0$, and $\:{\lambda\:}_{edge}=4.0$, the model records FID 188.95. In Phase II, $\:{\lambda\:}_{L1}$is reduced to 5.0, $\:{\lambda\:}_{FM}$to 10.0, and $\:{\lambda\:}_{SSIM}$to 0.35, while the remaining loss weights are preserved. By epoch 16, corresponding to the first checkpoint evaluated under the Phase II weight schedule, FID improves substantially to 85.80. Continued optimization under the Phase II schedule further improves performance, with the converged full model reaching FID 59.67. These observations suggest that strong early reconstruction and feature-alignment supervision stabilize initial synthesis, while reduced reconstruction weighting later in training permits the adversarial and perceptual objectives to refine high-frequency chromogenic structure.

Among the individually ablated auxiliary losses, Sobel Edge Loss exerts the strongest influence on perceptual realism. Removing the edge-consistency term ($\:{\lambda\:}_{edge}=0$) increases FID from 59.67 to approximately 88.0 (+ 47.5%) and degrades LPIPS from 0.527 to 0.539. This indicates that neither pixel-wise reconstruction nor perceptual supervision alone provides sufficient gradient emphasis for preserving high-frequency tissue edges and structural boundaries. By contrast, removing the DAB consistency losses ($\:{\lambda\:}_{DAB1}={\lambda\:}_{DAB2}=0$) raises FID to approximately 68.3 (+ 14.5%) and increases LPIPS to approximately 0.531, demonstrating that chemically grounded stain-space supervision contributes to perceptual fidelity beyond what adversarial and reconstruction losses alone can provide.

Several loss terms included in the full model were not individually ablated. Some of these losses are directly associated with dedicated architectural components, including the feature matching loss used with the multi-scale discriminator, the contrastive alignment loss associated with the Feature Alignment Block, and the auxiliary deep supervision loss applied to the auxiliary prediction branch. Other terms, including SSIM, hematoxylin consistency, color consistency, and variance regularization, operate as output-level supervision or regularization objectives within the overall training framework. Their isolated contributions therefore remain unquantified in the present study and may interact with both the phased weight schedule and the ablated loss terms reported in Table 5.

**Table 5:** Loss function and phased weight-schedule ablation on the BCI dataset

Config	PSNR↑	SSIM↑	LPIPS↓	FID↓	ΔFID (%)
**Checkpoint at end of Phase I (epoch 15):** $\:{\boldsymbol{\lambda\:}}_{\boldsymbol{L}1}=8.0$, $\:{\boldsymbol{\lambda\:}}_{\boldsymbol{L}\boldsymbol{P}\boldsymbol{I}\boldsymbol{P}\boldsymbol{S}}=12.0$, $\:{\boldsymbol{\lambda\:}}_{\boldsymbol{F}\boldsymbol{M}}=15.0$, $\:{\boldsymbol{\lambda\:}}_{\boldsymbol{S}\boldsymbol{S}\boldsymbol{I}\boldsymbol{M}}=0.6$, $\:{\boldsymbol{\lambda\:}}_{\boldsymbol{D}\boldsymbol{A}\boldsymbol{B}2}=10.0$, $\:{\boldsymbol{\lambda\:}}_{\boldsymbol{e}\boldsymbol{d}\boldsymbol{g}\boldsymbol{e}}=4.0$	18.34	0.518	0.575	188.95	+ 216.7
**Checkpoint at start of Phase II (epoch 16):** $\:{\boldsymbol{\lambda\:}}_{\boldsymbol{L}1}=5.0$, $\:{\boldsymbol{\lambda\:}}_{\boldsymbol{L}\boldsymbol{P}\boldsymbol{I}\boldsymbol{P}\boldsymbol{S}}=12.0$, $\:{\boldsymbol{\lambda\:}}_{\boldsymbol{F}\boldsymbol{M}}=10.0$, $\:{\boldsymbol{\lambda\:}}_{\boldsymbol{S}\boldsymbol{S}\boldsymbol{I}\boldsymbol{M}}=0.35$, $\:{\boldsymbol{\lambda\:}}_{\boldsymbol{D}\boldsymbol{A}\boldsymbol{B}2}=10.0$, $\:{\boldsymbol{\lambda\:}}_{\boldsymbol{e}\boldsymbol{d}\boldsymbol{g}\boldsymbol{e}}=4.0$	20.00	0.478	0.533	85.80	+ 43.8
**Full model w/o Sobel Edge (** $\:{\boldsymbol{\lambda\:}}_{\boldsymbol{e}\boldsymbol{d}\boldsymbol{g}\boldsymbol{e}}=0$ **)**	21.20	0.464	0.539	88.0	+ 47.5
**Full model w/o DAB Consistency (**$\:{\boldsymbol{\lambda\:}}_{\boldsymbol{D}\boldsymbol{A}\boldsymbol{B}1}$ = $\:{\boldsymbol{\lambda\:}}_{\boldsymbol{D}\boldsymbol{A}\boldsymbol{B}2}=0$**)**	21.90	0.471	0.531	68.30	+ 14.5
**Full model (phased schedule: **$\:{\boldsymbol{\lambda\:}}_{\boldsymbol{L}1}=8\to\:5$, $\:{\boldsymbol{\lambda\:}}_{\boldsymbol{L}\boldsymbol{P}\boldsymbol{I}\boldsymbol{P}\boldsymbol{S}}=12.0$, $\:{\boldsymbol{\lambda\:}}_{\boldsymbol{F}\boldsymbol{M}}=15\to\:10$, $\:{\boldsymbol{\lambda\:}}_{\boldsymbol{S}\boldsymbol{S}\boldsymbol{I}\boldsymbol{M}}=0.6\to\:0.35$, $\:{\boldsymbol{\lambda\:}}_{\boldsymbol{e}\boldsymbol{d}\boldsymbol{g}\boldsymbol{e}}=4.0$, $\:{\boldsymbol{\lambda\:}}_{\boldsymbol{D}\boldsymbol{A}\boldsymbol{B}2}=10.0$**)**	**22.50**	**0.477**	**0.527**	**59.67**	**—**

ΔFID is relative to the full model. ↑ higher is better; ↓ lower is better

Overall, the ablation results indicate that realism-oriented supervision and structure-preserving inductive biases contribute more substantially to performance than architectural depth alone.

### Evaluation using an independent HER2 classifier

To further evaluate whether the synthesized virtual IHC images preserve HER2 score–related information, an independent ConvNeXt classifier was trained exclusively on real HER2 IHC images from the BCI dataset and evaluated on both real and synthesized images without any retraining. On the held-out real IHC test set, the classifier achieved an accuracy of 94.78%, a macro F1-score of 95.24%, and a quadratic-weighted Cohen’s κ of 0.9423. When applied directly to a subset of the synthesized virtual IHC images generated by the proposed framework, the classifier achieved an accuracy of 72.07%, a macro F1-score of 70.78%, and a quadratic-weighted Cohen’s κ of 0.6190 (Table 6). Although a performance reduction relative to real IHC images is expected because of the domain gap between real and synthesized images, these results indicate that the generated virtual IHC images retain substantial HER2 score–discriminative information rather than merely reproducing the visual appearance of IHC staining.

**Table 6:** Performance of an independent ConvNeXt classifier on real and synthesized virtual HER2 IHC images

Test Set	Accuracy (%)	Macro F1-score (%)	Quadratic-weighted Cohen’s κ
Real IHC	94.78	95.24	0.9423
Generated Virtual IHC	72.07	70.78	0.6190

### Failure cases and limitations

**Fig. 16:**
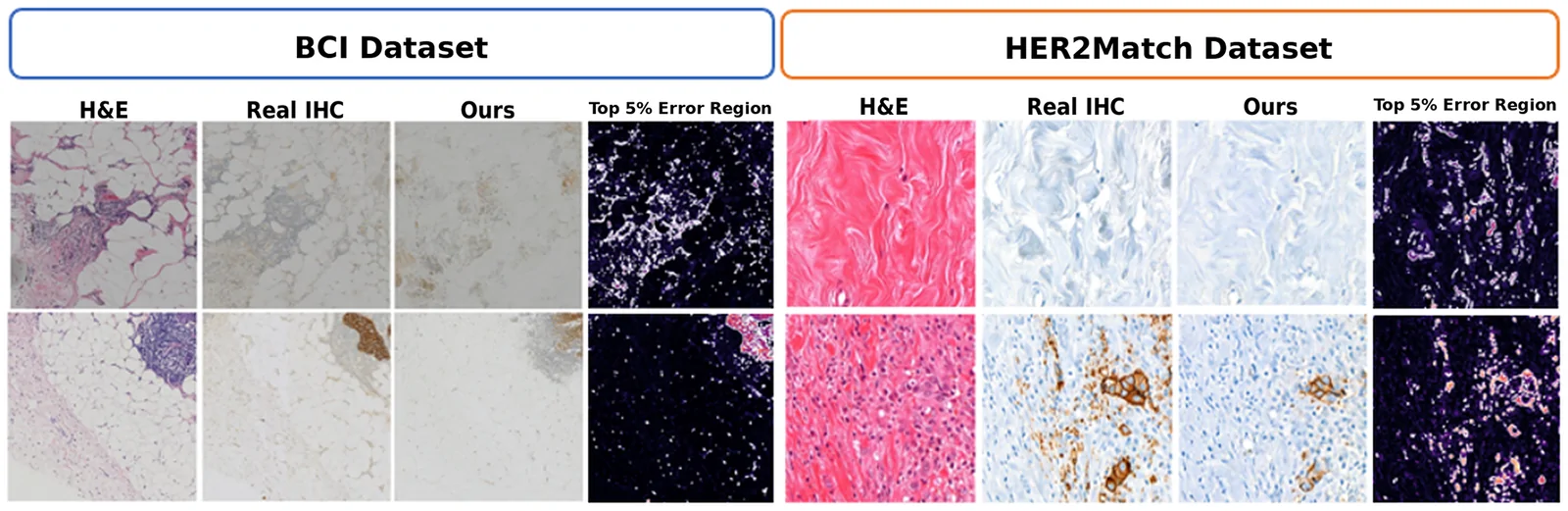
Representative failure cases on the BCI and HER2match datasets. From left to right: H&E, ground-truth IHC, synthesized IHC, and the corresponding top 5% pixel-wise error region. Errors are primarily localized to strongly stained membrane structures

Although the proposed framework achieves strong overall quantitative and qualitative performance, certain challenging regions remain difficult to synthesize accurately. Figure 16 presents representative failure cases from the BCI and HER2match datasets together with the corresponding top 5% error regions. In the error maps, **brighter colors indicate larger reconstruction errors**, whereas darker regions correspond to lower error.

As illustrated in Fig. 16, the largest errors are primarily concentrated around regions with strong membrane staining, sharp chromogenic transitions, and complex glandular structures, which contain fine spatial details and high local intensity variation. In contrast, relatively homogeneous tissue regions generally exhibit lower reconstruction error. Compared with HER2match, the BCI examples show more spatially extended error regions, consistent with the greater variability introduced by adjacent-section image pairs, whereas the HER2match errors are more localized due to the same-slide paired acquisition protocol.

Beyond these representative examples, several limitations remain. First, the proposed framework is designed for **paired H&E–IHC training data**, where corresponding H&E and IHC images are spatially aligned. Although the two-stage architecture improves robustness to moderate registration inaccuracies, its applicability to fully unpaired datasets remain to be investigated. Second, the current implementation is specifically optimized for **HER2** virtual staining; extending the framework to other immunohistochemical biomarkers, such as ER, PR, or Ki67, will likely require adapting both the stain-prior representation and the conditioning mechanism. Finally, while the proposed framework achieves strong quantitative performance and realistic visual synthesis, its clinical utility has not yet been validated through prospective pathologist reader studies. Future work will address these limitations by extending the framework to additional biomarkers, investigating unpaired virtual staining, performing large-scale multi-center clinical validation, and evaluating the framework across more diverse clinical cohorts and staining protocols.

## Conclusion

This work presents a HER2-score-conditioned two-stage GAN framework for synthesizing virtual HER2 IHC images from routine H&E histology sections. By decomposing the translation process into stain-prior prediction and refinement stages with hierarchical HER2-score conditioning, the proposed architecture addresses key challenges in computational virtual staining, including consistent stain-component generation, and effective propagation of biomarker-conditioning signals. Comprehensive experiments on the BCI and HER2match benchmarks demonstrate state-of-the-art distributional and perceptual performance across multiple quantitative metrics, while qualitative comparisons and Integrated Gradients attribution analysis confirm that the refinement stage actively exploits the predicted stain-prior channels as designed.

Generated virtual IHC images may support secondary digital pathology applications, including stain standardization across laboratories, educational dataset generation, retrospective computational pathology studies, and controlled counterfactual visualization of HER2 expression patterns when the biomarker status is already known.

Future work will focus on extending the framework to additional immunohistochemical biomarkers (ER, PR, Ki67) through biomarker-specific stain priors and conditioning mechanisms. Investigating unpaired virtual staining settings would broaden applicability beyond aligned H&E–IHC datasets. Additionally, we will explore whether high-fidelity synthetic IHC images can serve as augmented training data for downstream HER2 scoring classifiers, though such predictive capability would require a separate model architecture and extensive molecular ground-truth validation. Prospective pathologist reader studies remain essential to assess whether the perceptual improvements captured by FID, LPIPS, and KID translate into clinically meaningful image quality.

Collectively, the proposed framework provides a robust and interpretable foundation for computational virtual immunohistochemistry synthesis, supporting future research toward standardized, reproducible, and scalable AI-assisted digital pathology applications.

## Data Availability

Data availability: BCI dataset: https://github.com/bupt-ai-cz/BCI/blob/main/download_dataset.md HER2match Dataset: https://zenodo.org/records/15797050.
